# Exploration of immunological responses underpinning severe fever with thrombocytopenia syndrome virus infection reveals IL-6 as a therapeutic target in an immunocompromised mouse model

**DOI:** 10.1093/pnasnexus/pgac024

**Published:** 2022-03-10

**Authors:** Steven R Bryden, James I Dunlop, Andrew T Clarke, Mazigh Fares, Marieke Pingen, Yan Wu, Brian J Willett, Arvind H Patel, George F Gao, Alain Kohl, Benjamin Brennan

**Affiliations:** Medical Research Council–University of Glasgow Centre for Virus Research, Glasgow G61 1QH, Scotland, UK; Medical Research Council–University of Glasgow Centre for Virus Research, Glasgow G61 1QH, Scotland, UK; Medical Research Council–University of Glasgow Centre for Virus Research, Glasgow G61 1QH, Scotland, UK; Medical Research Council–University of Glasgow Centre for Virus Research, Glasgow G61 1QH, Scotland, UK; Institute of Infection, Immunity and Inflammation, College of Medical, Veterinary and Life Sciences, University of Glasgow, Glasgow G12 8TA, UK; Department of Pathogen Microbiology, School of Basic Medical Sciences, Capital Medical University, Beijing 100069, China; Medical Research Council–University of Glasgow Centre for Virus Research, Glasgow G61 1QH, Scotland, UK; Medical Research Council–University of Glasgow Centre for Virus Research, Glasgow G61 1QH, Scotland, UK; CAS Key Laboratory of Pathogen Microbiology and Immunology, Institute of Microbiology , Chinese Academy of Sciences (CAS), Beijing 100101, China; Medical Research Council–University of Glasgow Centre for Virus Research, Glasgow G61 1QH, Scotland, UK; Medical Research Council–University of Glasgow Centre for Virus Research, Glasgow G61 1QH, Scotland, UK

**Keywords:** SFTS, emerging bandavirus, pathogenesis, animal model, therapy

## Abstract

*Dabie bandavirus* (previously severe fever with thrombocytopenia syndrome virus; SFTSV), is an emerging tick-borne bunyavirus responsible for severe fever with thrombocytopenia syndrome (SFTS), a disease with high case fatality that is characterized by high fever, thrombocytopenia, and potentially lethal hemorrhagic manifestations. Currently, neither effective therapeutic strategies nor approved vaccines exist for SFTS. Therefore, there remains a pressing need to better understand the pathogenesis of the disease and to identify therapeutic strategies to ameliorate SFTS outcomes. Using a type I interferon (IFN)-deficient mouse model, we investigated the viral tropism, disease kinetics, and the role of the virulence factor nonstructural protein (NSs) in SFTS. Ly6C^+^ MHCII^+^ cells in the lymphatic tissues were identified as an important target cell for SFTSV. Advanced SFTS was characterized by significant migration of inflammatory leukocytes, notably neutrophils, into the lymph node and spleen, however, these cells were not required to orchestrate the disease phenotype. The development of SFTS was associated with significant upregulation of proinflammatory cytokines, including high levels of IFN-γ and IL-6 in the serum, lymph node, and spleen. Humoral immunity generated by inoculation with delNSs SFTSV was 100% protective. Importantly, NSs was critical to the inhibition of the host IFNɣ response or downstream IFN-stimulated gene production and allowed for the establishment of severe disease. Finally, therapeutic but not prophylactic use of anti-IL-6 antibodies significantly increased the survival of mice following SFTSV infection and, therefore, this treatment modality presents a novel therapeutic strategy for treating severe SFTS.

Significance Statement *Dabie bandavirus* (formerly severe fever with thrombocytopenia syndrome virus), is an emerging viral pathogen of widening concern, first discovered in 2009. It is a tick-borne bunyavirus responsible for causing an often-fatal disease called severe fever with thrombocytopenia syndrome (SFTS). Currently, no vaccines or antiviral therapies have been licenced for use. In this report, we use recombinant viruses to elucidate the host immunological responses that occur in advanced SFTS. We show that the disease was characterized by migration of neutrophils to tissues and upregulation of several cytokines, notably IFN-γ and IL-6. NSs is critical to the inhibition of the host IFNɣ response during infection. Finally, we demonstrate that therapeutic use of anti-IL-6 antibodies significantly increased the survival of mice following SFTSV infection.

## Introduction

*Dabie bandavirus*, formerly severe fever with thrombocytopenia syndrome virus (SFTSV), is an emerging tick-borne *Phenuivirus* (genus *Bandavirus* (1)) of increasing medical concern for which no specific antiviral drugs or effective vaccines have been approved. First described in Eastern China in 2009 but with serological evidence reported as early as 1996 (2), SFTSV has since increased rapidly in both incidence and geographical range, causing over 13,000 reported human cases in China (2010–2019) (3), 3,137 cases in South Korea (2013–2017), 303 cases in Japan (4) with other serological surveys detecting SFTSV in tick populations in Vietnam, Taiwan, and Pakistan (5). SFTSV typically causes a nonspecific febrile illness—severe fever with thrombocytopenia syndrome (SFTS) with symptoms ranging from fever, malaise, myalgia, arthralgia, to thrombocytopenia, and leukopenia (6). Occasionally, SFTS presents clinically as a severe hemorrhagic fever with symptoms including cerebral hemorrhage, gastrointestinal bleeding, and multiple organ failure (7). In 2018, SFTSV was declared a priority pathogen by the World Health Organisation (WHO) due to a lack of effective medicines and a case mortality rate estimated between 5% and 30% (3). We propose that further defining the key cellular and molecular mechanisms that underpin SFTS is important for identifying novel therapeutic targets to ameliorate severe disease outcomes.

SFTSV has a tri-segmented single-stranded negative or ambisense RNA genome consisting of the Large (L), Medium (M), and Small (S) segments. The L segment encodes the RNA-dependent RNA polymerase (RdRp), the M segment encodes the viral glycoproteins (Gn and Gc), and the S segment encodes the nucleocapsid protein (N) and a nonstructural protein (NSs) (8). Many published studies have now shown the NSs protein to be crucial in the antagonism of the mammalian innate immune response and NSs has been identified as a key virulence factor, reviewed in Khalil et al (9). SFTSV NSs can sequester and spatially isolate key antiviral innate immune molecules into virus-derived inclusion bodies. These molecules include critical aspects of the host type I Interferon (IFN) response and viral pattern recognition receptors including: STAT1, STAT2, IRF3, IRF7, and RIG-I (9). Consequently, a recombinant SFTSV lacking NSs (published as rHB2912aaNSs (10); herein referred to as delNSs SFTSV) has been shown to result in limited pathogenicity and induce a robust humoral response in an aged ferret model of disease, suggesting a potential role for this recombinant virus as a live-attenuated vaccine candidate (11). Despite the promising data obtained in the aged ferret model, little is known regarding the host cellular and molecular immune mechanisms that lead to the reduction in severity of disease observed following infection with this virus. It is, therefore, of vital importance that these mechanisms are elucidated and understood to ensure the safety of any such live-attenuated vaccine candidates and to understand the pathogenic processes associated with both wild-type (wt) and delNSs SFTSV.

In humans, advanced SFTS i.e. requiring hospitalization, is generally associated with high levels of proinflammatory cytokines in the serum, including IFNɣ, IL-6, IL-1α, TNF, and inflammatory CC and CXC chemokines including CCL3, CCL4, and CXCL8 (12, 13). In SFTSV-infected patients, a “cytokine storm” is considered the main pathophysiological feature of severe and fatal disease along with hemorrhagic complications arising from thrombocytopenia. Such cytokine storming is a phenomenon that is often associated with other viral hemorrhagic fevers such as Ebola virus disease (14) and more recently with severe cases of SARS-CoV-2 infection (15). Further understanding of the inflammatory milieu within the host may lead to the identification of therapeutic targets that could limit the damage induced by aberrant cytokine storms.

There are two ways of targeting viral immunopathology, either virus replication is targeted or the inflammatory response to viral infections must be dampened. As such, therapeutic interventions for bunyaviral disease are rare, and for SFTS there are no clinically proven treatments available for infected patients (16–18). Treatment with ribavirin has shown variable results in case studies and animal models of infection (19). Studies in patients showed ribavirin was ineffective as a treatment option if the viral load in the patient was greater than 1 × 10^6^ copies/ml (19). The efficacy of Favipiravir has also been demonstrated in vivo, with 100% survival of animals if treated within 3–4 days of initial infection (20, 21). A small-scale clinical study has demonstrated that a 5-day course of Favipiravir treatment led to a decline in SFTSV viral load in two patients experiencing different clinical manifestations (22). Importantly, both drugs target viral replication rather than prevent the cytokine storming associated with severe manifestations of SFTS (13, 23–25).

In this study, we take a two-pronged approach to examining SFTS disease. First, we have developed and characterized a murine model of SFTSV to investigate the tissue tropism, cellular tropism, and clinical progression of disease. This allowed us to elucidate additional roles for SFTSV NSs in helping to establish an infection in vivo in our small animal model. These new pathogenesis studies were conducted with a live-attenuated vaccine candidate delNSs SFTSV. Second, using this robust model we demonstrate that therapeutic administration of an anti-IL-6 antibody led to a reduction in disease severity and increased survival of lethally challenged mice; thereby, proposing a treatment option that does not directly target virus replication. These important data show the potential for modulation of host cellular and inflammatory responses as a future therapeutic strategy to ameliorate SFTS in human patients.

## Results

### SFTSV NSs determines disease severity in an *IFNAR^−^^/^^−^* mouse model of infection

**Fig. 1.**
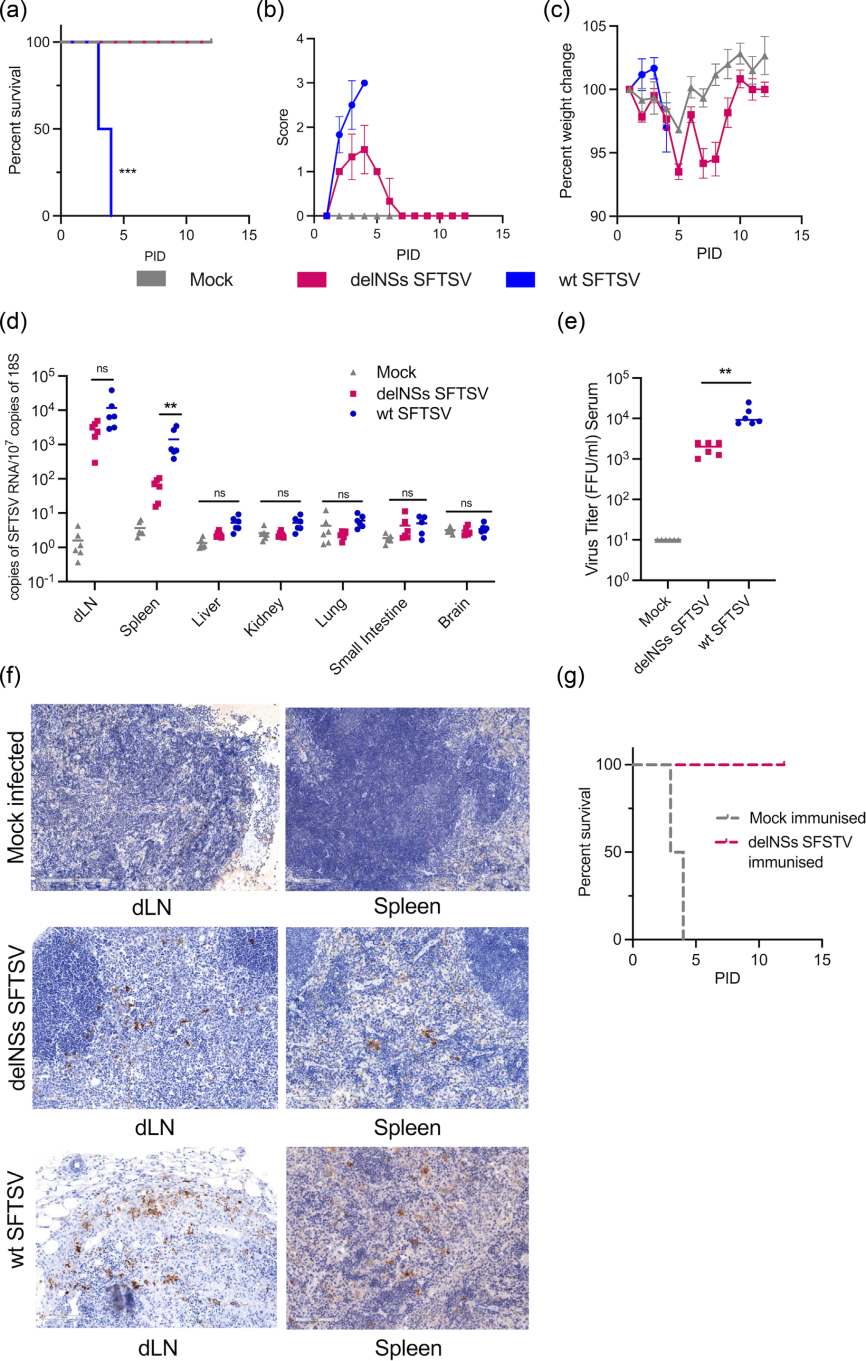
Development of a IFNAR *^−^^/^^−^* animal model for SFTS disease. Groups of six A129 *IFNAR^−^^/^^−^* mice were inoculated with 10^5^ foci-forming units (FFU) of wt or delNSs SFTSV- or mock-infected. The survival (a), clinical scores (b), or weight change (c) were monitored at the indicated times postinfection. (d) Distribution and viral RNA copy number in tissues of SFTSV-infected mice. Tissues from mice infected with delNSs SFTSV (pink), wt SFTSV- (blue), or mock-infected (gray) were collected at PID 3 and viral copy numbers were assessed by real-time PCR. Examined tissues were draining lymph node (dLN), spleen, liver, kidney, lung, small intestine, and brain. (e) Viral titre was assessed in the serum of infected animals at PID 3 by foci-forming assay. (f) Immunohistochemical staining of SFTSV-infected tissues. dLNs and spleens of infected (delNSs or wt SFTSV) or mock animals were harvested at PID 3 and reacted with a SFTSV anti-N antibody. (g) Groups of six A129 *IFNAR^−^^/^^−^* mice were immunized with 10^5^ FFU of delNSs SFTSV or mock immunized. Subsequently, animals were challenged with a known lethal dose of wt SFTSV and monitored for disease progression and survival. Asterisks indicated significance **P < 0.01, ***P < 0.001, or ns = not significant as measured by a log-rank (Mantel–Cox) test (a), Kruskal–Wallis test with Dunn's multiple comparison test (d), or a Mann–Whitney test (e).

To investigate the tropism and pathogenesis of SFTSV and to elucidate the role of NSs in determining disease severity, we first characterized an in vivo murine model that supported viral replication and presented clinical signs of SFTS. Previous murine models have utilized new-born immunocompetent mice, the use of putative immune suppressants or IFN alpha receptor knockout (*IFNAR^−^^/^^−^*) mice (26). To assess the ability of adult, immunocompetent wt mice to support SFTSV replication, 8-week-old wt C57BL/6 mice, or 8-week-old C57BL/6 human STAT 2 knock-in (hSTAT2 KI) mice were infected with 10^5^ focus-forming units (FFU) of the HB29 strain of SFTSV (either wt or delNSs). hSTAT2 KI mice were selected due to previous literature suggesting SFTSV can suppress antiviral immunity via sequestering of human, but not murine STAT2 (27). At 3 days postinfection (dpi), no virus could be detected in either the serum nor in any of the tissues sampled (Supplementary Material Appendix, Figure S1), leading us to hypothesize that SFTSV is either unable to infect these animals or cannot sufficiently evade the murine innate immune response, and hence establish an infection, even in the presence of human STAT2. Next, the suitability of 8-week-old A129 *IFNAR^−^^/^^−^* mice as a model of SFTS was investigated by infecting the mice with 10^5^ FFU of either wt or delNSs SFTSV, then measuring weight and scoring clinical signs. All mice infected with wt SFTS reached a clinically defined endpoint by postinfection day (PID) 3–4, whereas all mice infected with delNSs SFTSV recovered from infection (Fig. 1a). All *IFNAR^−^^/^^−^* mice infected with wt SFTSV developed signs of disease and clinical signs including subdued behavior when stimulated, piloerection, hunching, eye discharge, and mild weight loss (< 10%) and were humanely killed by PID 4, when predetermined endpoints were reached (Fig. 1b and c). Importantly, *IFNAR^−^^/^^−^* mice infected with delNSs SFTSV developed only mild, transient disease (Fig. 1b) with mild weight loss (< 10%) that was fully recovered (Fig. 1c). While it is known that the virulence factor NSs is often associated with type I IFN antagonism and is a known virulence factor also for other bunyaviruses, here, NSs is revealed as a determinant of disease severity, even in a murine system lacking a type I IFN response.

After developing a model that presented a disease phenotype for wt SFTSV infection, and which confirmed the attenuated qualities of delNSs SFTSV as a vaccine candidate, we next investigated the tissue tropism of both viruses in in vivo studies. Eight-week-old A129 *IFNAR^−^^/^^−^* mice were infected with 10^5^ FFU of either wt or delNSs SFTSV. Mice were euthanized at PID 3 (when signs of disease became apparent in wt SFTSV-infected mice) and tissues were sampled. SFTSV RNA was detected by qPCR in the draining inguinal lymph node (dLN) adjacent to the injection site and in the spleen, but not in remote, nonlymphatic tissues for either delNSs SFTSV or wt SFTSV (Fig. 1d). wt SFTSV RNA was readily detectible in the draining lymph node (dLN) and was also found to be present at a statistically higher copy number than that of delNSs SFTSV RNA in the spleen (*P <* 0.01; Fig. 1d). The higher levels of RNA detected in wt SFTSV-infected animals is likely a result of the higher viraemia detected in the blood, as detected by viral foci forming assays (Fig. 1e and Supplementary Material Appendix, Figure S1a). The tropism of SFTSV was further confirmed by immunohistochemical staining for SFTSV N protein in these tissues (Fig. 1f). We confirmed that virus was present in both the dLN and the spleen of animals infected with either wt or delNSs SFTSV, with greater staining being observed in the wt SFTSV-infected tissues (Fig. 1f). In addition, we investigated the pathology of infected tissues through hematoxylin and eosin (H&E) staining of the spleen and lymph node at PID 3. Here, delNSs SFTSV infection resulted in minimal disruption of the tissue architecture, however, wt SFTSV infection resulted in prominent disruption of the white pulp of the spleen compared to spleens isolated from mock-infected mice (Supplementary Material Appendix, Figure S2).

The delNSs SFTSV has been suggested previously as a live-attenuated vaccine for the induction of protective immunity in an aged ferret model of SFTS. Due to the reduced virulence of the delNSs SFTSV in *IFNAR^−^^/^^−^* mice, we next investigated the ability of delNSs SFTSV to induce protective immunity in our immunocompromised model. *IFNAR^−^^/^^−^* mice were infected with a single dose of 10^5^ FFU delNSs SFTSV and monitored for clinical signs and virus titre in the serum. Viraemia peaked at PID 3 and was followed by a drop in virus titre at PID 4 (*P <* 0.05; Supplementary Material Appendix, Figure S3a). From this point on, mice regained weight and clinical scoring returned to below a mild threshold. Surviving mice were then subsequently infected with 10^5^ FFU of wt SFTSV at PID 14, a dose known to be lethal from previous studies (Fig. 1a). Importantly, this single dose of attenuated virus resulted in 100% survival of the immunized and challenged animals to PID 12, compared to 0% survival in the mock immunized group, with all animals reaching humane endpoints by PID 4 (Fig. 1g). Passive transfer of purified IgG from recovered delNSs SFTSV-infected mice also conferred 100% protection from a lethal challenge dose of wt SFSTV, indicating that humoral immunity was sufficient for protection (Supplementary Material Appendix, Figure S3b).

### SFTSV preferentially infects CCR2^+^, Ly6C^+^ cells in lymphatic tissues

**Fig. 2.**
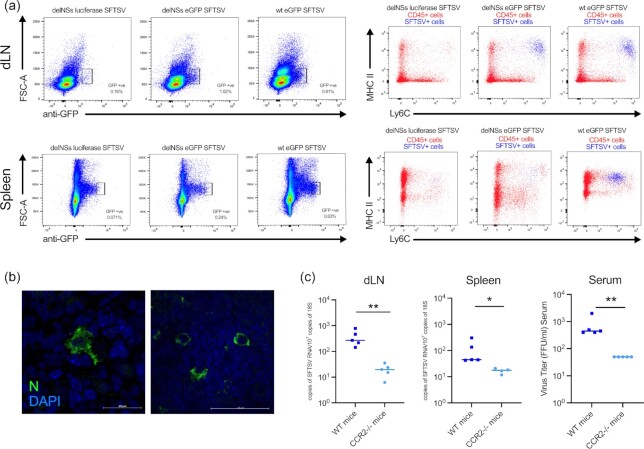
Cellular tropism of SFTSV in infected mice. Groups of six A129 *IFNAR^−^^/^^−^* mice were infected with SFTS viruses expressing reporter proteins or wt SFTSV. Reporter viruses included examples where the NSs ORF had been replaced with either humanized luciferase (delNSs luciferase SFTSV) or eGFP (delNSs eGFP SFTSV) and a virus where eGFP has been C-terminally fused to the NSs ORF (wt eGFP SFTSV). (a) Draining inguinal lymph nodes and spleens were collected from mice infected with 10^5^ FFU of delNSs luciferase SFTSV, delNSs eGFP SFTSV, or wt eGFP SFTSV at PID 3 and processed for flow cytometry. Initial analysis was performed against forward scatter (FSC-A) and eGFP expression. Subpopulations of FSC-A/GFP positive cells were further analyzed for expression of MHC class II and Ly6C. (b) Spleen of *n* = 2 mice infected with wt SFTSV were harvested at PID 3, fixed, embedded, and sectioned, and reacted with an antibody to SFTSV N protein (green) and DAPI (blue). Images show representative staining of cells showing myelomonocytic morphology within the spleen. (c) Groups of five CCR2 *^−^^/^^−^* mice or wt C57BL/6 were infected with 10^5^ FFU of wt SFTSV. At PID 3, mice were killed, and the dLN and spleen were harvested to examine for the presence of viral RNA. Serum from the infected animals was also collected to assay for the presence of virus by immunofocus assay. Asterisks indicated significance *P < 0.05 or **P < 0.01 as measured by a Mann–Whitney test (c).

To further delineate the mechanisms of SFTSV-induced pathogenesis, we next investigated the cellular tropism of the virus in vivo, an understanding of which could lead to cell specific, therapeutic targeting. *IFNAR^−^^/^^−^* mice were infected with either 10^5^ FFU of a recombinant SFTSV expressing a NSs protein that is fused in-frame to enhanced green fluorescent protein (eGFP; wt eGFP SFTSV) or a recombinant virus in which the NSs open reading frame (ORF) was replaced entirely by eGFP (delNSs eGFP SFTSV) (10). A virus expressing humanized *Renilla* luciferase (delNSs luciferase SFTSV) was used as a negative control for eGFP virus infection (10). Mice were killed at PID 3, and cells were stained for both GFP–APC to identify infected cells, and cell surface markers, which facilitated identification of immune cells. Stained cells were then analyzed by flow cytometry (Fig. 2a). In the splenic and lymphatic tissues of wt and delNSs eGFP SFTSV-infected mice, the majority of anti-GFP (APC) stained cells were positive for CD45, Ly6C, and MHCII. In contrast, this population was not detected in the tissues of the delNSs luciferase SFTSV-infected control mice (Fig. 2a). These data suggest that monocytic cells are a key target of SFTSV infection. Furthermore, these flow cytometry data confirmed the reduced dissemination of NSs-deletant viruses to distal tissues such as the spleen. Immunofluorescence microscopy was conducted from splenic tissue harvested from wt SFTSV-infected mice. These data additionally revealed target cells with myelomonocytic morphology, supporting the data obtained by flow cytometry (Fig. 2b).

To confirm the contribution of myelomonocytic cells to SFTSV tropism and viral dissemination, groups of C57BL/6 CCR2*^−^^/^^−^* mice that had their IFN response repressed with anti-IFN antibodies (24-hours prior to infection; PID −1) were inoculated with 10^5^ FFU of wt SFTSV. As previously described (28), CCR2*^−^^/^^−^* mice have a greater than 90% reduction of peripheral myelomonocytic cells, including Ly6C^+^ cells. Tissues were harvested and assayed at PID 3, and significantly less viral RNA was detected in the lymph node and splenic tissues of CCR2*^−^^/^^−^* mice compared to wt C57BL/6 controls animals. The reduced detection of viral RNA in these tissues was mirrored by the reduced number of virus particles detected in the blood of wt SFTSV-infected CCR2*^−^^/^^−^* mice, as evidenced through immunofocus assays (Fig. 2c). The data suggest that CCR2^+^ cells (which include the inflammatory Ly6C^+^ cells we identified as key cellular targets of SFTSV infection [Fig. 2a]), were important for establishing SFTSV infection of mice.

### wt SFTSV infection causes thrombocytopenia and drives migration of neutrophils and monocyte-derived cells

**Fig. 3.**
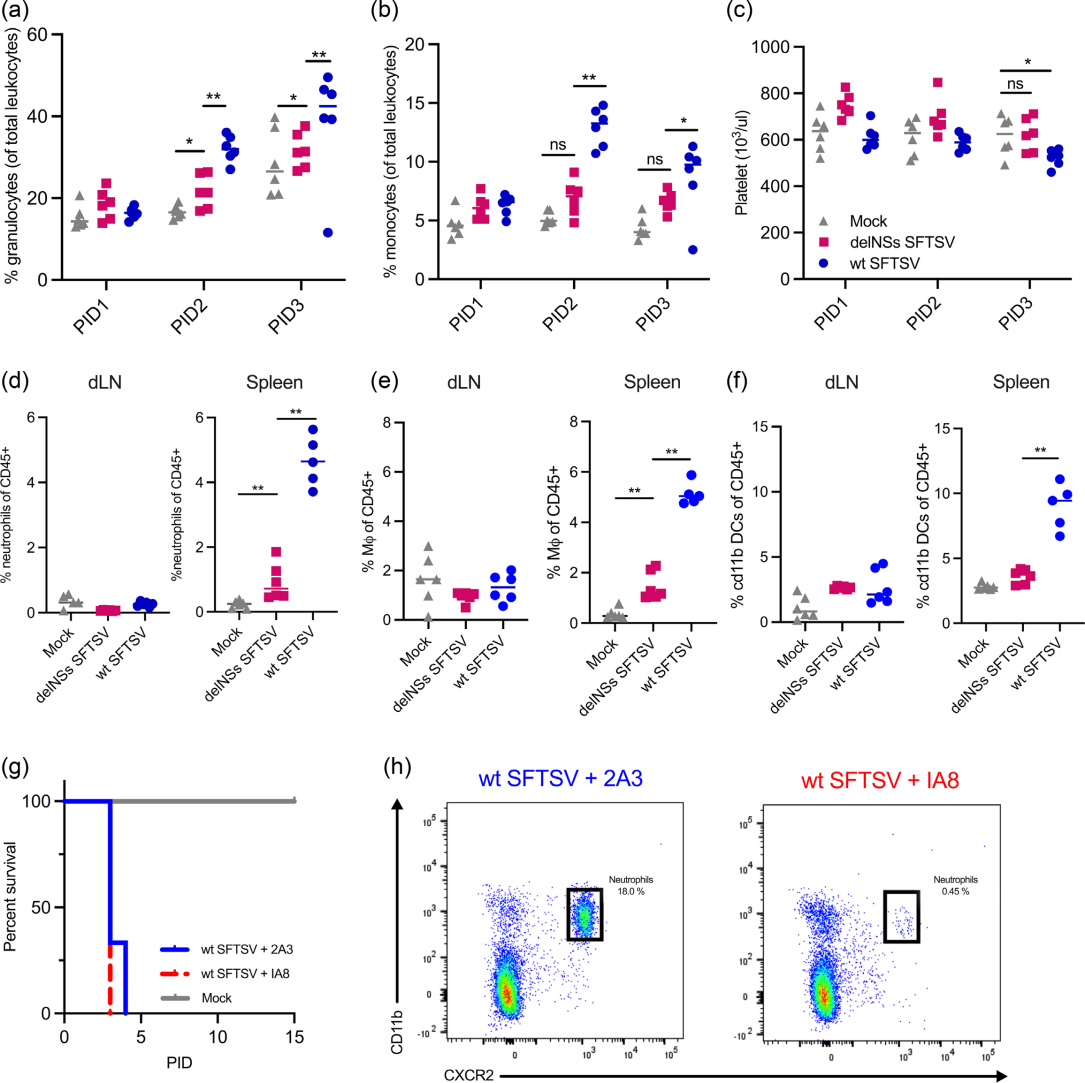
Infection causes thrombocytopenia and neutrophilia in mice, but neutrophilia is not responsible for the pathogenic phenotype observed in wt SFTSV-infected animals. Groups of six A129 *IFNAR^−^^/^^−^* mice were inoculated with 10^5^ foci-forming units (FFU) of wt (blue) or delNSs SFTSV (pink) or mock-infected (gray). At the indicated times postinfection, blood was taken for hematological analysis of % granulocytes of total leukocytes (a), % monocytes of total leukocytes (b), or estimation of platelet levels (c). At PID 3, dLN and spleen of infected animals were processed for flow cytometry to analyze the levels of neutrophils (d), monocytes (e), or DCs (f) in the tissues. (g) Survival curve of infected mice. Groups of six A129 *IFNAR^−^^/^^−^* mice had neutrophils depleted at PID −3 and PID −1 through the administration of an anti-Ly6 [1A8; red] antibody or an isotype control antibody [2A3; blue], all animals were then infected with wt SFTSV- or mock-infected (gray). Infection was allowed to progress until humane endpoints were reached. (h) Neutrophil depletion demonstrated by flow cytometric analysis (CD11b vs. CXCR2) of PID 3 blood samples taken from isotype control (blue) or neutrophil depleted (red) wt SFTSV-infected animals. Neutrophil population is indicated in the black box. Asterisks indicated significance *P < 0.05, **P < 0.01, or ns = not significant as measured by a Mann–Whitney test (a)–(f).

Previous longitudinal studies in patients admitted to hospital with severe or lethal SFTS, demonstrated that the major pathophysiological features of SFTSV infection are cytokine-mediated inflammation and the hemorrhagic manifestations resulting from thrombocytopenia (25, 29, 30). To elucidate further the contribution of thrombocytopenia to SFTS pathology, the levels of circulating platelets and the cellular composition of inflammatory leukocytes were measured after infection with either wt or delNSs SFTSV. Mice were infected with 10^5^ FFU of wt SFTSV, delNSs SFTSV or vehicle control. Blood samples were taken daily and analyzed on a medical hematology analyzer, while tissues were harvested at the conclusion of the experiment on PID 3. Cells within the tissues were then stained for cell surface markers and analyzed by flow cytometry (Fig. 3). Analysis of the cellular composition in the blood demonstrated that virulent wt SFTSV infection resulted in a significant increase in granulocytes over avirulent delNSs SFTSV infection from PID 2 onward, despite delNSs SFTSV infection also promoting a significant increase in granulocytes in the blood over mock-infected animals (Fig. 3a). Additionally, wt SFTSV infection promoted monocyte chemotaxis into the blood, with significant increases in blood monocytes observed on PID 2 and PID 3 (Fig. 3b). Blood analysis also provided evidence of a small, but statistically significant (*P* = 0.0411) reduction in peripheral platelet numbers at PID 3 in the blood of wt SFTSV-infected animals compared to delNSs SFTSV infection or mock-infected controls (Fig. 3c). When the dLN and spleen were examined at PID 3, flow cytometric analysis demonstrated that both wt and delNSs SFTSV infection resulted in significant increases of CXCR2^+^, Ly6G^+^, and CD11b^+^ neutrophils in the spleen but not in the dLN (Fig. 3d). Additionally, as with blood granulocytes, splenic neutrophilia was observed to be significantly higher in the virulent wt virus infection compared to the avirulent delNSs vaccine candidate infection (Fig. 3d). wt SFTSV infection also resulted in statistically significant increases in CD11b^+^, CD64^+^ macrophages (Mφ; Fig. 3e) and CD11c^+^, CD64^−^ dendritic cells (DCs; Fig. 3f) in the spleen compared to mock-infected or delNSs SFTSV-infected mice.

To confirm if the observed neutrophilia contributed to the pathogenicity of wt SFTSV in A129 *IFNAR^−^^/^^−^* mice, animals were depleted of neutrophils by administration of an anti-Ly6G (1A8) antibody, or mock-depleted by administration of an isotype-matched control (2A3), at PID −3 and PID −1. Mice were then inoculated with 10^5^ FFU of wt SFTSV or mock-infected and were monitored for the development of clinical signs. When predetermined end points were reached, animals were then killed humanely, and blood was taken for flow cytometric analyses. No difference in virulence was observed in neutrophil-depleted mice compared to those treated with the isotype-matched control antibody and all mice in both groups reached humane end points on either PID 3 or PID 4 (Fig.   3g). Flow cytometric analysis confirmed that the depletion of neutrophils was successful and that neutrophils remained absent following infection, this was confirmed by a reduction in the CD11b^+^/CXCR2^+^ population of cells from 18% in the blood of isotype control animals to 0.45% in depleted animals (Fig. 3h). These data demonstrate conclusively that while the neutrophilia observed in the blood of wt SFTSV-infected animals is correlated with severe disease, neutrophils are not the cause of the observed pathogenic phenotype in this model.

### Lethal SFTSV infection is associated with increases in inflammatory cytokines IL-1, IL-12, IL-6, and IFNɣ

**Fig. 4.**
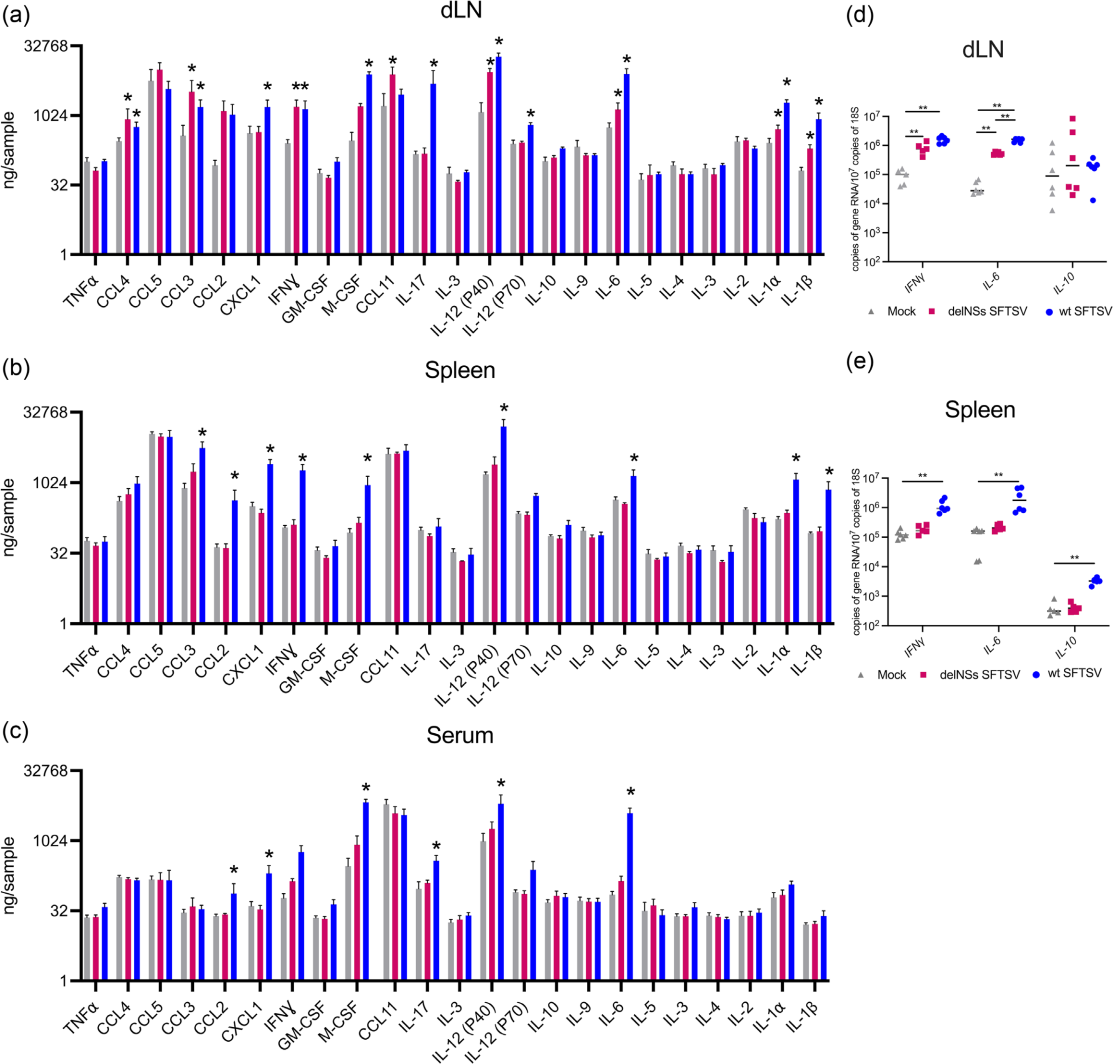
Cytokine and chemokine analysis of infected mouse tissues and serum. Groups of four A129 *IFNAR^−^^/^^−^* mice were inoculated with 10^5^ foci-forming units (FFU) of wt (blue) or delNSs SFTSV- (pink) or mock-infected (gray). At PID 3, mice were killed, and the dLN, spleen, and serum were collected from the mice for cytokine and chemokine profiling by Luminex assay or quantitative PCR analysis. Luminex assay panel for dLN (a), spleen (b), or serum (c) showing the differential detection of a panel of proteins involved in immune signaling. Graphs are shown comparing protein abundance in mock-infected (gray), delNSs SFTSV-infected (pink), or wt SFTSV-infected tissues. Quantitative PCR was performed to examine differential expression of *IFNɣ, IL-6*, and *IL-10* genes from mice that were mock-infected (gray), delNSs SFTSV-infected (pink), or wt SFTSV-infected. DLN (d) or spleen (e) were examined. Asterisks indicated significance *P < 0.05, **P < 0.01, or ns = not significant as measured by a Mann–Whitney test (a)–(e).

After defining the tissue and cellular tropism of SFTSV in vivo and further elucidating the cellular immune profile of infected tissues, next we analyzed cytokine production in infected tissues to further delineate the mechanism of SFTS disease. These increases were then confirmed by selective qPCR assays (Fig. 4). In these experiments, mice were infected with 10^5^ FFU of wt SFTSV, delNSs SFTSV or vehicle control (DMEM). At PID 3, lymph node (Fig. 4a), spleen (Fig. 4b), and serum (Fig. 4c) were taken from mice for Luminex and qPCR analysis (Fig. 4d and e). A total of 23 cytokines were analyzed, many of which were upregulated significantly during either wt or delNSs SFTSV infection compared to samples from mock-infected animals. The inguinal lymph node displayed the largest upregulation of inflammatory cytokines with CCL4, CCL3, CXCL1, IFNɣ, M-CSF, CCL11, IL-17, IL-12(P40), IL-12 (P70), IL-6, IL-1⍺, and IL-1β all being recorded at levels significantly higher than controls. IL-17 was the most upregulated cytokine in the dLN, being recorded at an amount 31-fold greater in the lymph node samples from wt SFTSV-infected animals compared to those from mock-infected animals (Fig 4a). IFNɣ was significantly upregulated in the lymph node (Fig. 4a and d) and spleen (Fig. 4b and e) of wt SFTSV-infected mice, but not in paired serum samples (Fig. 4c) at PID 3. CXCL1, M-CSF, and IL-12 (P40) and IL-6 were significantly upregulated in all 3 sample types assayed from wt SFTSV-infected animals. Of note was IL-6, as this proinflammatory cytokine was upregulated an average 14-fold in the lymph node, 3-fold in the spleen, and 56-fold in the serum of wt SFTSV-infected animals (*n* = 4). No significant increase in any cytokine included in the Luminex assay was observed in the spleen (Fig. 4b) or serum (Fig. 4c) of delNSs SFTSV-infected animals. This may reflect the poor dissemination of the virus to distal tissues of the infected mice as reported earlier in the study.

### IFNɣ is required for protection from delNSs SFTSV infection

**Fig. 5.**
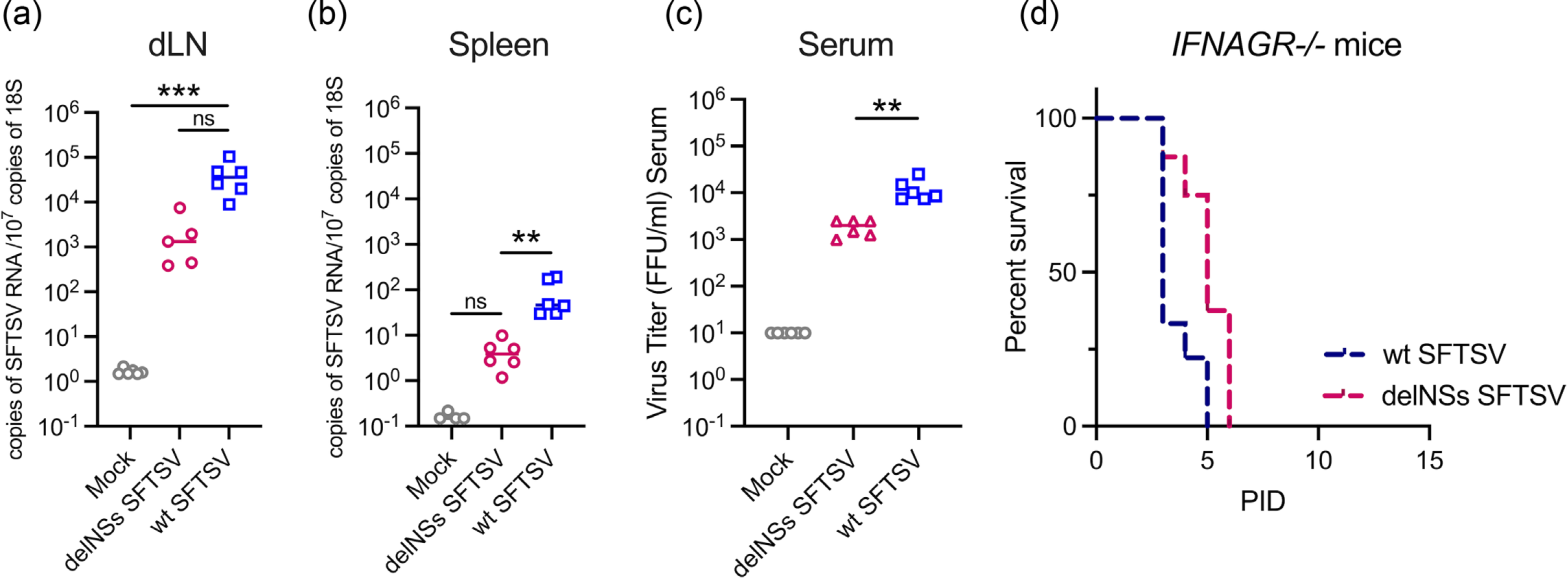
Pathogenesis of recombinant SFTS viruses in *IFNAGR^−^^/^^−^* mice. Groups of six AG129 *IFNAGR^−^^/^^−^* mice were mock-infected (gray) or inoculated with 10^5^ foci-forming units (FFU) of delNSs (pink) or wt SFTSV (blue). At PID 3, dLN (a), spleen (b), or serum (c) samples from infected animals were collected and viral copy numbers were assessed by real-time PCR. (d) Survival curve of infected *IFNAGR^−^^/^^−^* mice. Groups of six AG129 *IFNAGR^−^^/^^−^* mice were infected with 10^5^ foci-forming units (FFU) of wt SFTSV (blue) or delNSs SFTSV (pink) and infection was allowed to progress until clinical signs were observed and humane endpoints were reached. Asterisks indicated significance **P < 0.01, ***P < 0.001, or ns = not significant as measured by a Kruskal–Wallis test with Dunn's multiple comparison test (a)–(c).

IFNɣ is a key antiviral cytokine that has been described previously to exhibit strong anti-SFTSV activity (31). Importantly, in our Luminex and qPCR screen, IFNɣ was upregulated in the dLN of both wt and delNSs SFTSV-infected mice (Fig. 4a) and in the spleen of wt SFTSV-infected mice (Fig. 4b). We, therefore, hypothesized that the NSs protein of wt SFTSV was important for modulating the host IFNɣ response, allowing efficient dissemination of the virus to the spleen, where the virus then triggered a deleterious inflammatory response. To investigate this hypothesis, groups of AG129 IFNαβɣ receptor knockout (*IFNAGR^−^^/^^−^*) mice were infected with 10^5^ FFU of wt SFTSV or delNSs SFTSV. Tissues and sera were sampled from the infected mice at PID 3, the presence of viral RNA was quantified via qPCR, and viraemia was determined by viral immunofocus assay. The viral titers of both wt SFTSV and delNSs SFTSV in *IFNAGR^−^^/^^−^* mice were similar to those observed in our previous experiments using A129 *IFNAR^−^^/^^−^* mice (Fig. 1), despite the lack of a functional IFNɣ response (Fig. 5). No difference was observed in the replication of either virus in the dLN of infected *IFNAGR^−^^/^^−^* mice (Fig. 5a). Further, delNSs SFTSV did not disseminate to the spleen of infected *IFNAGR^−^^/^^−^* mice as effectively as wt SFTSV (Fig. 5b) and mice infected with the attenuated virus demonstrated a statistically significant (*P* = 0.0290) reduction in infectious virus in the serum (Fig. 5c). We next investigated the role of IFNɣ in defining the clinical outcome of disease for both wt and delNSs SFTSV-infected animals. Groups of *IFNAGR^−^^/^^−^* mice were infected with 10^5^ FFU of either wt SFTSV or delNSs SFTSV. Mice were then monitored for clinical signs then humanely killed at clinically defined endpoints. As observed previously with the *IFNAR^−^^/^^−^* mice (Fig. 1a), all *IFNAGR^−^^/^^−^* mice infected with wt SFTSV reached clinical endpoints at or before PID 5. Interestingly, in the absence of IFNɣ signaling, mice infected with the avirulent delNSs SFTSV developed clinical signs of infection and reached humane endpoints at or before PID 6 (Fig. 5d). These data strongly indicate a pivotal role for IFNɣ in the protection of mice from delNSs SFTSV infection and suggest that the observed in vitro interactions of SFTSV NSs and components of the type II IFN pathway (31) may form the basis of a relationship between NSs and the modulation of host IFNɣ responses in vivo.

### Therapeutic, but not prophylactic, treatment with an anti-IL-6 antibody significantly improves survival of *IFNAR^−^^/^^−^* mice to wt SFTSV infection

**Fig. 6.**
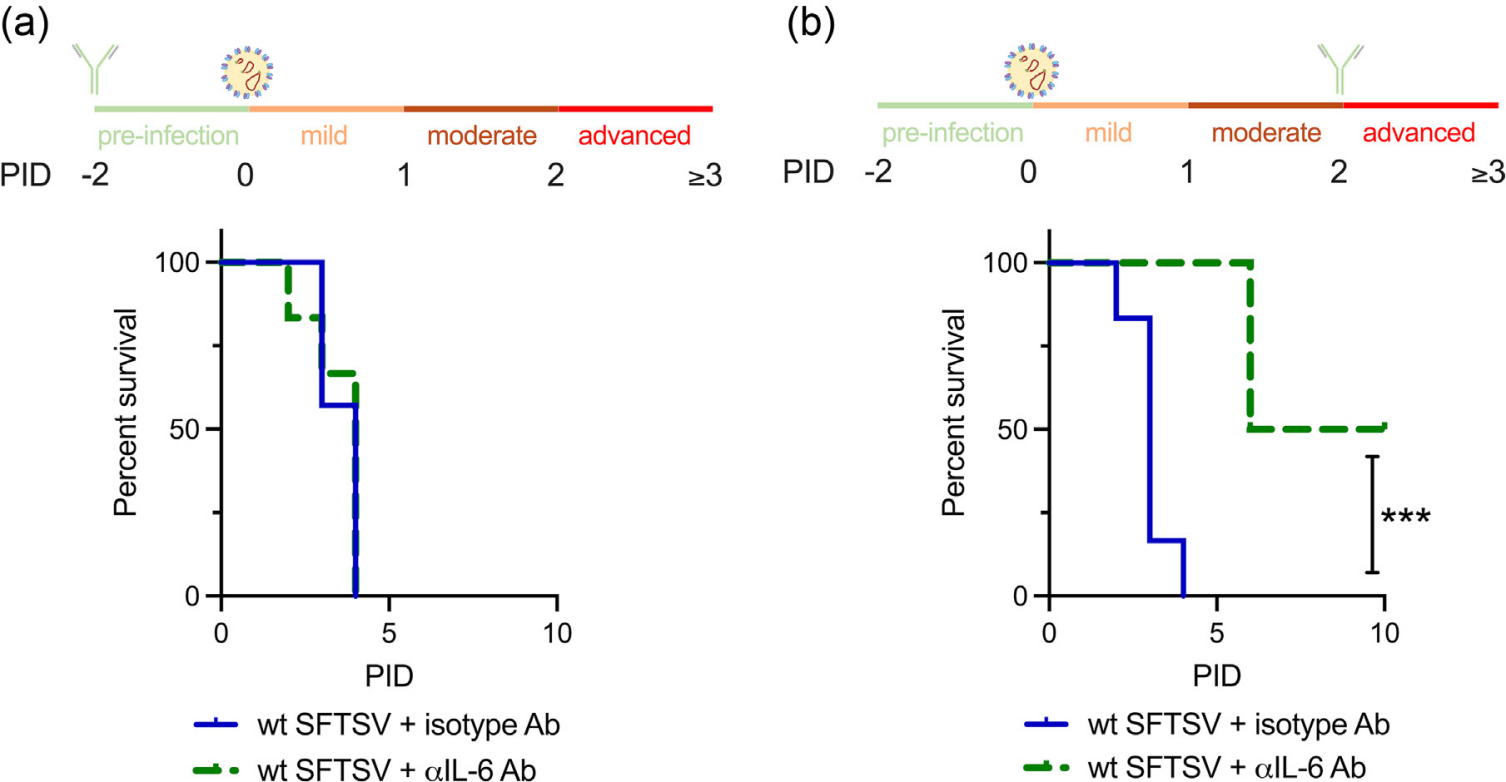
Therapeutic not prophylactic administration of an anti-IL-6 antibody decreases clinical signs and increased survival of wt SFTSV-infected mice. Groups of six A129 *IFNAR^−^^/^^−^* mice were interperitoneally administered an isotype control antibody (blue) or an anti-IL-6 antibody (green), either prophylactically at PID −2 (a) or therapeutically at PID 2 (b). All animals in all groups were inoculated with 10^5^ foci-forming units (FFU) of wt SFTSV at PID 0 and infection was allowed to progress until clinical signs were observed and humane endpoints were reached. Asterisks indicated significance ***P < 0.001 as measured by a log-rank (Mantel–Cox) test.

IL-6 is an important mediator of fever and acute phase response to inflammation and has been documented to act as both a proinflammatory cytokine and an anti-inflammatory myokine (reviewed in (32)). IL-6 also acts as an important checkpoint in the regulation of neutrophil trafficking during inflammatory responses, causing further chemokine production. Given the statistically significant increase in IL-6 levels observed in the dLNs, spleens, and sera of wt SFTSV-infected mice (Fig. 4a–c), we next examined whether prophylactic or therapeutic administration of an anti-IL-6 antibody could improve the clinical course of disease observed in the infected mice. Groups of A129 *IFNAR^−^^/^^−^* mice were infected with 10^5^ FFU of wt SFTSV, and administered either an anti-IL-6 antibody (2B Scientific; BE0046) or an isotype control antibody (BioXCell; BP0089) at PID −2 (prophylactic, before infection; Fig.   6a) or at PID 2 (therapeutic, after infection; Fig. 6b). Clinical scoring for the mice used in Fig. 6 can be found in Supplementary Material Appendix, Figure S6. PID 2 was chosen, as this is the time point at which mice display moderate clinical signs in our model when infected with wt SFTSV (Fig. 1b).

Following infection with wt SFTSV, all mice were monitored, and clinical signs were observed until humane endpoints or PID 10 was reached. There was no observable difference in disease severity or survival between the anti-IL-6 treated mice or the control mice when the antibody treatment was administered preinfection (PID −2), with all mice reaching predetermined end points by PID 4 (Fig. 6a).

Following this result, we hypothesized that the lack of efficacy of prophylactic anti IL-6 treatment in preventing severe SFTS could be due to critical antiviral functions of IL-6 during the early stages of infection. Indeed, it has previously been demonstrated that IL-6 is required for protection against several viruses including influenza and vaccinia viruses during the early stages of infection (33). We, therefore, attempted to block IL-6 therapeutically at PID 2, when moderate clinical signs become apparent and when dysregulation of the inflammatory response may be causing excessive, potentially lethal damage to the organs. Here, groups of animals were infected with 10^5^ FFU of wt SFTSV, and subsequently at PID 2, IL-6, or isotype control antibodies were administered. This therapeutic administration of anti-IL-6 not only doubled the survival time (from PID 3 to PID 6) of the lethally challenged, but it also resulted in 50% of the animals surviving and recovering from infection, clearing all clinical signs by PID 10.

## Discussion

SFTSV is an emerging tick-borne bunyavirus of significant medical concern that is associated with substantial morbidity and mortality. Despite this, no specifically approved medicines or vaccines are currently available to treat SFTSV infection or ameliorate the potentially lethal symptoms of SFTS-mediated disease. One of the major challenges in developing new therapeutic strategies for SFTSV, is the limited characterization of the current small animal models that replicate key aspects of severe human disease. Indeed, there is now a pressing need to investigate the mechanisms that underlie the pathogenesis of SFTSV in a model that reconstitutes lethal disease, to identify potential therapeutic targets. Several animal models have been described for recapitulating SFTS disease, summarized in a recent publication by Matsuno et al. (26). Various mouse strains, hamster, rat, and macaque models have been trialled to understand the pathogenesis of tickborne phenuiviruses. However, most models described do not result in lethal infection and disease like those displayed in infected humans. A few groups have previously described the use of IFNAR-/- mice in the study of SFTSV pathogenesis (20, 26, 34, 35). This study is the first to describe and dissect the underlying mechanisms of pathogenesis of recombinant virulence factor knock out SFTS viruses. In this study, we developed a highly tractable murine model of lethal SFTSV infection in immunocompromised A129 *IFNAR^−^^/^^−^* mice. We characterized the tissue and cellular tropism of SFTSV (strain HB29) in vivo, elucidated the role of the NSs in contributing to severe disease, and investigated the immunopathogenic mechanisms defining lethal SFTS. Following on from this basic characterization of our model, we examined the potential of a previously described live-attenuated vaccine candidate (delNSs SFTSV) that engendered 100% survival in our model and, as a proof of principle, modulated the host inflammatory response by therapeutically depleting IL-6 at PID 2 when animals are starting to display advanced clinical signs, leading to significantly increased survival of lethally challenged animals. This manuscript therefore describes the complete development of a robust small animal model, through to the exploration of potential antiviral therapies.

The pathogenicity and tropism of wt SFTSV and a delNSs SFTSV vaccine candidate were compared in *IFNAR^−^^/^^−^* mice. Wt SFTSV infection resulted in a rapid onset severe disease, with all mice succumbing to infection within 4 dpi. Interestingly, delNSs SFTSV infection resulted in a mild transient disease in which all mice recovered. These findings contrast previous studies in which a NSs-deletant Bunyamwera orthobunyavirus (36), or two attenuated Rift Valley fever phlebovirus strains (37), displayed similar clinical signs and lethality as the related wt viruses in an IFNAR*^−^^/^^−^* mouse model of infection. These data demonstrate that compared to other NSs-deletant bunyaviruses, our delNSs SFTSV is particularly attenuated. Using a type I IFN deficient mouse model, we have shown that SFTSV NSs does not confer virulence exclusively through the widely accepted mechanism; via modulation of the host type I IFN response. Due to the avirulent nature of delNSs SFTSV, including within immunocompromised mice, we next confirmed its ability to induce a protective adaptive immune response. Here, all mice challenged with wt SFTSV at 14 days postinoculation with delNSs SFTSV survived the lethal challenge. Additionally, we demonstrated that humoral immunity induced by delNSs SFTSV was sufficient for protection from lethal challenge through transfer of purified IgG from delNSs SFTSV-infected mice to naïve mice prior to challenge with wt SFTSV. This, in tandem with previous studies showing the induction of sterilizing immunity in aged ferrets following inoculation with delNSs SFTSV (11), demonstrates the potential for this virus to be further developed as a live-attenuated vaccine candidate.

After defining the disease progression of both viruses and further demonstrating the potential of delNSs SFTSV as a vaccine candidate, we next investigated the tissue and cellular tropism of both viruses. We found that lymphatic tissues were susceptible to infection with both viruses, whereas nonlymphatic tissues (i.e. liver, kidney, lungs, brain, and gut) remained refractory. An important finding was that, whereas both wt and delNSs SFTSV RNA were found in the dLN in similar quantities, SFTSV RNA was significantly lower in delNSs SFTSV-infected spleens compared to those seen in wt SFTSV-infected animals, and less infectious units were measured in the blood via plaque assay. This is despite both viruses having similar replication kinetics in IFN-deficient cell lines in vitro (10). These data suggest that a type I IFN independent innate antiviral response can inhibit viral replication or dissemination in the absence of NSs. Subsequently, it was demonstrated that delNSs SFTSV virulence could be restored by removing the type II IFN (IFNɣ) response, suggesting that IFNɣ is critical for controlling delNSs SFTSV infection, and that NSs can inhibit the action of the IFNɣ pathway or downstream IFNɣ-stimulated genes to confer virulence, hinting at another unexplored role for SFTSV NSs in viral pathogenesis that requires future investigation.

In our model of infection, both wt and delNSs SFTSV-infected Ly6C^+^, MHCII^+^ myelomonocytic cells preferentially. These cells are bone marrow derived and migratory, and as such may represent a means by which SFTSV disseminates. By using mice that were peripherally deficient in these cells, *CCR2^−^^/^^−^* mice treated with anti-IFNAR antibodies, we demonstrated that wt SFTSV replication was significantly impaired at the draining LN and reduced replication in and dissemination to the spleen and the serum were noted, compared to infected wt mice (also treated with anti-IFNAR antibodies). Moreover, infection of GM-CSF differentiated bone marrow cells and primary skin fibroblasts in vitro provided further evidence of the susceptibility of leukocytes to infection, while fibroblasts remained refractory (Supplementary Material Appendix, Figure S4). These findings are intriguing, as an important stage in the establishment of infection for most arboviruses is thought to be the seeding of virus in the fibroblastic cells of the skin, before subsequent infection of leukocytes, enabling dissemination to the lymph nodes (38–40). However, it is important to note here, that the virus was delivered subcutaneously by injection and not through the bite of an infected arthropod. The injection method may not be able to reach the same early target cells as those accessed during a tick bite. Future work will be undertaken to address this question directly. Importantly, in the days following wt SFTSV infection, the myeloblastic derived cells, which are preferentially infected, are found in significant numbers in both the dLN and the spleen. Immediately after infection with wt SFTSV, neutrophils influx into the LN and spleen. Granulocytes (mostly neutrophils) were detected in high numbers in the blood, as well as monocytes, DCs and macrophages, all of which were also significantly increased in lymph node and spleen of infected animals. Importantly, migration of these cell types to the spleen was significantly lower in mice infected with delNSs SFTSV. Reduced migration of potentially infected cells could explain the lower viral burden in the spleen of mice infected with this attenuated virus. Alternatively, limited delNSs SFTSV replication may result in the lower amounts of cytokine and chemokines, leading to reduced cell migration to in the distal tissues.

Another key feature described here, was the association of neutrophilia and severe disease. It has often been hypothesized that neutrophils may orchestrate the inflammatory environment that leads to pathogenesis and disease progression in some viral infections (41). To ascertain whether this was the case for SFTSV, we selectively depleted neutrophils during the early phase of infection. Importantly, despite the close association between disease progression, disease severity and neutrophils in the blood, the removal of neutrophils had no impact on the clinical outcome of infection with wt SFTSV. These data indicate that neutrophils, although potentially contributory to pathogenesis, are dispensable for the progression of SFTS. The influx of neutrophils into the sera or tissues may be caused by the dysregulation of inflammatory mediators such as IL-17 that were observed in wt SFTSV-infected mice (42, 43). Despite this finding, neutrophilia remains a key prognostic indicator between mild and advanced disease and could be used to stratify patients clinically into those requiring more urgent therapeutic or medical interventions, such the treatment with Ribavirin or Favipiravir.

It has been demonstrated in many clinical studies that upregulation of proinflammatory cytokines in human SFTS patients is strongly correlated with a poor disease outcome (13, 25, 30, 44). This was reflected in our mouse model, where we found a notable upregulation of the proinflammatory cytokines IL-12, IL-6, IL-1⍺, IL-1β, and IFNɣ. IFNɣ and IL-6 have been described to be key indicators of disease severity in patients with mild vs. severe SFTS (45, 46). As we have shown here, IFNɣ is indispensable for protection against avirulent delNSs SFTSV infection. IFNɣ is potentially a major target of NSs, and therefore, may be considered “protective” in the context of SFTSV infection. Another key antiviral cytokine that is thought to contribute to the pathogenesis of other viral-induced inflammatory diseases is Interleukin-6 (IL-6). IL-6 has been implicated as a pathogenic factor in the lungs of SARS-CoV-2 patients, and clinical trials involving anti-IL-6 biologics have shown promise (47). Due to the relationship between IL-6 and lethal pathology in viral infections, and due to the range of approved anti-IL-6 drugs available on the market for diseases including rheumatoid arthritis and multiple sclerosis (such as the IL-6 receptor antagonists tocilizumab and sarilumab or drugs that act directly on IL-6, sirukumab). We next decided to investigate the impact of depleting IL-6 both prophylactically, or therapeutically, in SFTSV-infected mice. Perhaps unsurprisingly, due to the importance of IL-6 in controlling the early stages of viral infections the prophylactic use of anti-IL-6 antibody had no impact on survival of mice infected with wt SFTSV. Interestingly, therapeutic injection of anti-IL-6 antibody at the point where signs of disease progress from mild to moderate (PID 2) increased the survival of infected mice significantly. This has important consequences clinically for cases of human SFTS. Indeed, the point at which patients are admitted to hospital with symptomatic SFTS (7–14 days, typically around 9 days following a tick bite), is analogous to the point at which disease progresses from mild to moderate in mice, after which the virus has replicated and disseminated. We suggest that anti-IL-6 antibody treatment using repurposing of clinically approved drugs could improve the clinical outcome of SFTS and should now be investigated as a potential therapeutic strategy for advanced cases of SFTS. Such treatments could be efficacious in late stages of the disease in humans, particularly around the time symptoms have caused the patient to seek medical intervention. It is important to note that this treatment strategy targets the host response to viral infection and can, therefore, be beneficial in late stages of disease when active virus replication may be waning, and direct targeting of the virus is less efficacious.

Such immunopathologies are observed during infection with other pathogenic bunyaviruses such as *Crimean Congo hemorrhagic fever orthonairovirus* (48) and *Rift Valley fever phlebovirus* (49), and aberrant cytokine production is reported to be a contributing factor to disease phenotypes observed. This work, therefore, highlights the potential for the modulation of immune responses as a novel therapeutic modality to treat the plethora of bunyaviral diseases that affect both animal and human populations.

## Methods

### Mice

Unless otherwise specified, all mice were 8–12-week-old IFN alpha receptor knockout (*IFNAR^−^^/^^−^*) mice on a 129S7/SvEvBrdBkl-Hprt^b-m2^ background (Marshall Bioresources), comprised of both male and female animals. Human Stat 2 knock in mice (hSTAT2 KI; C57BL/6-*Stat2^tm1.1(STAT2)^^Diam^*/AgsaJ) (50) were kindly provided by Professor Adolfo Garcia-Sastre, Icahn School of Medicine at Mount Sinai. A129 (*IFNAR^−^^/^^−^*), AG129 (*IFNAGR^−^^/^^−^*), hSTAT2 KI ,and CCR2*^−^^/^^−^* mice were derived from a locally bred colony and maintained in a pathogen-free facility, in filter-topped cages, and maintained in accordance with local and governmental regulations. hSTAT2 KI, A129 (*IFNAR^−^^/^^−^*), and AG129 (*IFNAGR^−^^/^^−^*) deficient and WT counterparts were maintained in Techniplast 1284 L Blue line individually ventilated cages at Biological Services, University of Glasgow. Ccr2-deficient mice were originally obtained from the Jackson Laboratory (stock number 004999) (51). IFN alpha gamma receptor knockout (*IFNAGR^−^^/^^−^*) mice on a 129S7/SvEvBrdBkl-Hprt^b-m2^ background mice were originally purchased from Marshall Bioresources. All mice had a 12-hour light/12-hour dark cycle and were provided with sterile food and water.

### Animal ethics

All animal research described in this study was approved by the University of Glasgow Animal Welfare and Ethical Board and was carried out under United Kingdom Home Office Licenses, P9722FD8E, in accordance with the approved guidelines and under the UK Home Office Animals (Scientific Procedures) Act 1986 (ASPA). Mice were euthanized when they exhibited 3 or more signs of moderate severity or lost more than 15% body weight or at the indicated predefined endpoints. Scores assigned were as follows: 0, no clinical signs; 1, mild clinical signs apparent; 2, moderate clinical signs observable; and 3, 1 advanced clinical sign or 3 moderate clinical signs displayed. The clinical scoring table used in these studies can be found in Supplementary Material Appendix, Table S2.

### Cell culture

Vero E6 cell line was obtained from Institut Pasteur (provided by M. Bouloy) and grown in Dulbecco's modified Eagle's medium (DMEM) supplemented with 10% foetal calf serum (FCS), 10% tryptose phosphate broth, 100 units/ml penicillin, and 0.1 mg/ml streptomycin. Mouse DCs were derived from bone marrow precursors, using granulocyte colony-stimulating factor (GM-CSF; 20 ng/ml) for 6 days. Fibroblasts were derived from adult A129 (*IFNAR^−^^/^^−^*) dermis that was digested with collagenase D (1 mg/ml), dispase II (0.5 mg/ml), and deoxyribonuclease (DNase; 0.1 mg/ml) in Hank's balanced salt solution (HBSS) and grown DMEM supplemented with 10% FCS, 10% tryptose phosphate broth, 100 units/ml penicillin, and 0.1 mg/ml streptomycin.

### Viruses

SFTSV strains (rHB29pp [wt SFTSV] (52) or recombinant viruses rHB2912aaNSs [delNSs SFTSV], rHB29NSsGFP-FUSE [wt eGFP SFTSV], and rHB29delNSsGFP [delNSs eGFP SFTSV]; rHB29delNSshRen [delNSs luciferase SFTSV] (10)) were generated using reverse genetics technologies. This sequence is based on a plaque-purified stock called Hubei 29pp (HB29pp) provided by Amy Lambert (CDC Arbovirus Diseases Branch, Division of Vector-Borne Infectious Diseases, Fort Collins, CO). Working stocks of SFTSV were generated in a Vero E6 cell line by infecting at a low multiplicity of infection and harvesting the cell culture medium 7 dpi. Recombinant virus stocks were confirmed by Sanger sequencing to ensure no mutations had occurred during propagation. Previous data have demonstrated that recombinant viruses had the same replication kinetics and viral protein expression profiles as the wildtype/parental viruses in time course of infection studies (10, 52). Experiments with SFTSV were performed under containment level 3 (CL-3) conditions, approved by the UK Health and Safety Executive.

### Virus titration by immunofocus assays

Virus titres were determined by focus-forming assays in Vero E6 cells. Briefly, confluent monolayers of Vero E6 cells were infected with serial dilutions of virus made in phosphate buffer saline (PBS) containing 2% FCS and incubated for 1 hour at 37°C, followed by the addition of a GMEM overlay supplemented with 2% FCS and 0.6% Avicel (FMC Biopolymer). The cells were incubated for 6 days before fixation and subsequent use of focus-forming assays as described previously (10, 35).

### Infection of mice with viruses

Mice were anaesthetized by isoflurane inhalation and injected with 1 × 10^5^ foci-forming units (FFU) of SFTSV in 100 µl of DMEM, subcutaneously into the right flank using a 26G needle. Mice were monitored for moderate signs of infection and culled when they reached a clinically defined humane endpoint of disease or at specified timepoints.

### Flow cytometry

Single cell suspensions were acquired by passing spleens and lymph nodes through a 100 mm cell strainer. Cells were stained using a subset of antibodies and Fixable Viability Dye eFluor780 (eBioscience), fixed in Cytofix/Cytoperm (BD), and analyzed on a BD FACSAria III cell sorter (BD). Details of the gating strategies used in this study can be found in Supplementary Material Appendix, Figure S5.

### Depletion of neutrophils

To deplete neutrophils, mice were injected with antibodies that bind to Ly6G as per manufacturer's instructions (BioXcell). Mice were injected intraperitoneally with 200 μl of IA8 antibody or control antibody (2A3), which specifically depletes Ly6G^+^ cells, at 3 days and 1 day before infection. Successful neutrophil depletion was confirmed by flow cytometry analysis of peripheral blood using neutrophil markers CD11b^+^ and CXCR2^+^.

### RNA extraction from tissues

RNA was extracted using PureLink Plus columns and DNA digested on column as per manufacturer's instruction (Life technologies). Tissue samples were homogenized in TRIzol (Life Technologies) using a Precellys 24 (Bertin instruments) with 7 mm metal beads (Qiagen), followed by purification using PureLink columns with DNase digestion (Life Technologies).

### Gene expression analysis

Viral RNA and host gene transcripts were quantified by reverse transcription qPCR, and infectious virus was quantified by end point titration. Tissue generated up to 100 mg of total RNA, of which 1 mg of RNA was used to create cDNA, of which 1% was used per qPCR assay (10 ng of RNA equivalent). qPCR primers for SFTSV were designed to target the genomic M RNA. RNA was extracted using PureLink Plus columns and converted to cDNA using the High-Capacity RNA-to-cDNA kit (Life Technologies). qPCR analysis was undertaken using SYBR-green (Applied Biosystems) on a QuantStudio 7 Flex Real-Time PCR System (Applied Biosystems). Full details of primers used in this study can be found in Supplementary Material Appendix, Table S1.

### Blood analysis

Whole blood was collected and mixed with 10% 15 mg/ml tri-potassium EDTA. Blood analysis was undertaken on an ABX Micros ES 60 (Horiba Medical).

### Luminex assay

Tissues were homogenized in Bio-Plex® Cell Lysis buffer (Bio-Rad) containing protease inhibitors and clarified by centrifugation. Luminex assay was performed using Bio-Plex Pro Mouse Cytokine 23-plex Assay kit as per manufacturer's instructions (Bio-Rad) and analyzed on a Bio-Plex 200 (Bio-Rad).

### Indirect immunofluorescence staining

Co-cultures of DCs and fibroblasts were grown on coverslips. Once established, cells were infected with recombinant SFTS viruses at a moi of 0.1 FFU/cell. At 24 hours postinfection (pi), monolayers were fixed in 4% formaldehyde and were incubated with monospecific primary antibodies: rabbit antinucleocapsid SFTSV (52), CD11c (N418) eFluor 615 (eBioscience, #42–0114–82). Monolayers were then assayed with the following secondary antibodies: 1:500 Alexa Fluor 488 donkey antirabbit IgG antibody (Invitrogen). Slides were stained for cell nuclei using DAPI (4ʹ,6-diamidino-2-phenylindole) and mounted for viewing under the LSM 710 inverted confocal microscope in conjunction within Zen 3.2 Imaging software (Zeiss, Germany).

### Immunohistochemistry

Tissues were fixed in 4% methanol-free paraformaldehyde (Thermo Scientific) and then dehydrated in an increasing concentration of sucrose. Tissues were embedded in either optimal cutting temperature compound (Agar Scientific) or paraffin and sectioned. For immunohistochemistry cells were stained with anti-SFTSV N antibody by the University of Glasgow Veterinary diagnostics unit.

### Antibodies

A list of antibodies used in this study can be found in Supplementary Material Appendix, Table S3.

### Statistics

All in vivo mouse experiments were repeated a minimum of 2 times and a maximum of 3 times on separate occasions to confirm reproducibility of results. The exception to this is survival curves, which were undertaken either a single time (Figs 1g, 3g, 6, and Supplementary Material Appendix, Figure S3) or twice (all other survival curves), on ethical grounds in line with NC3Rs policy and the ARRIVE Guidelines 2.0 (53). In vivo experiments were performed with appropriate animal numbers to achieve a 90% power with a significance level (alpha) of 0.05 (2-tailed), calculated with StatMate 2.0 (GraphPad).

All in vitro studies and microscopy were undertaken on 3 separate occasions to ensure reproducibility. All results were confirmed between experimental replicates.

Data were analyzed using GraphPad Prism Version 9.2.0 software. Copy numbers of viral RNA and infectious titers from virus-infected mice were not normally distributed and were accordingly analyzed using nonparametric-based Mann–Whitney test or Kruskal–Wallis test with Dunn's multiple comparison test where appropriate, unless otherwise stated in figure legends. Survival curves were analyzed using the log-rank (Mantel–Cox) test. All plots have statistical significance: *P < 0.05; **P < 0.01; ***P < 0.001; ^****^P < 0.0001; and ns, not significant.

## Supplementary Material

pgac024_Supplemental_File

## Data Availability

The data that support the findings of this study are openly available from Enlighten Research Data at http://dx.doi.org/10.5525/gla.researchdata.1179. Authors will make reagents described in this study available on request (by qualified researchers for their own use). Requests should be directed to the corresponding author.

## References

[1] Abudurexiti A , et al. 2019. Taxonomy of the order Bunyavirales: update 2019. Arch Virol. 164:1949–1965. 31065850 10.1007/s00705-019-04253-6 PMC6641860

[2] Hu J . 2018. A cluster of cases of severe fever with thrombocytopenia syndrome bunyavirus infection in China, 1996: a retrospective serological study. PLoS NeglTrop Dis. 12:e0006603. 10.1371/journal.pntd.0006603 PMC6034904 29940000

[3] Yun SM , et al. 2020. Genetic and pathogenic diversity of severe fever with thrombocytopenia syndrome virus (SFTSV) in South Korea. JCI Insight. 5:e129531. 31877113 10.1172/jci.insight.129531 PMC7098714

[4] Kobayashi Y , et al. 2020. Severe fever with Thrombocytopenia Syndrome, Japan, 2013-2017. Emerg Infect Dis. 26:692–699. 32186502 10.3201/eid2604.191011 PMC7101122

[5] Zohaib A , et al. 2020. Serologic evidence of severe fever with thrombocytopenia syndrome virus and related viruses in Pakistan. Emerg Infect Dis. 26:1513–1516. 32568060 10.3201/eid2607.190611 PMC7323538

[6] Casel MA , Park SJ, Choi YK. 2021. Severe fever with thrombocytopenia syndrome virus: emerging novel phlebovirus and their control strategy. Exp Mol Med. 53:713–722. 33953322 10.1038/s12276-021-00610-1 PMC8178303

[7] Akagi K , et al. 2020. Detection of viral RNA in diverse body fluids in an SFTS patient with encephalopathy, gastrointestinal bleeding and pneumonia: a case report and literature review. BMC Infect Dis. 20:281. 32295538 10.1186/s12879-020-05012-8 PMC7160946

[8] Yu XJ , et al. 2011. Fever with thrombocytopenia associated with a novel bunyavirus in China. N Engl J Med. 364:1523–1532. 21410387 10.1056/NEJMoa1010095 PMC3113718

[9] Khalil J , Kato H, Fujita T. 2021. The role of non-structural protein NSs in the pathogenesis of severe fever with thrombocytopenia syndrome. Viruses. 13:876. 34064604 10.3390/v13050876 PMC8151429

[10] Brennan B , Rezelj VV, Elliott RM. 2017. Mapping of transcription termination within the S segment of SFTS Phlebovirus facilitated generation of NSs deletant viruses. J Virol.91:e00743–17. 28592543 10.1128/JVI.00743-17 PMC5533932

[11] Yu K-M , et al. 2019. Cross-genotype protection of live-attenuated vaccine candidate for severe fever with thrombocytopenia syndrome virus in a ferret model. Proc Natl Acad Sci. 116:26900–26908. 31818942 10.1073/pnas.1914704116 PMC6936527

[12] Seo JW , Kim D, Yun N, Kim DM. 2021. Clinical update of severe fever with thrombocytopenia syndrome. Viruses. 13:1213. 34201811 10.3390/v13071213 PMC8310018

[13] Deng B , et al. 2012. Cytokine and chemokine levels in patients with severe fever with thrombocytopenia syndrome virus. PLoS ONE. 7:e41365. 22911786 10.1371/journal.pone.0041365 PMC3404083

[14] Younan P , et al. 2017. Ebola virus binding to Tim-1 on T Lymphocytes induces a cytokine storm. mBio. 8:e00845–17. 28951472 10.1128/mBio.00845-17 PMC5615193

[15] Hojyo S , et al. 2020. How COVID-19 induces cytokine storm with high mortality. Inflammat Regenerat. 40:37. 10.1186/s41232-020-00146-3 PMC7527296 33014208

[16] Bopp NE . Baseline mapping of severe fever with thrombocytopenia syndrome virology, epidemiology and vaccine research and development. NPJ Vaccines. 5:111. 33335100 10.1038/s41541-020-00257-5 PMC7746727

[17] Takayama-Ito M , Saijo M. 2020. Antiviral drugs against severe fever with thrombocytopenia syndrome virus infection. Front Microbiol. 11:150. 32117168 10.3389/fmicb.2020.00150 PMC7026129

[18] Wu Y , et al. 2017. Structures of phlebovirus glycoprotein Gn and identification of a neutralizing antibody epitope. Proc Natl Acad Sci. 114:E7564–e7573. 28827346 10.1073/pnas.1705176114 PMC5594662

[19] Li H , et al. 2018. Epidemiological and clinical features of laboratory-diagnosed severe fever with thrombocytopenia syndrome in China, 2011-17: a prospective observational study. Lancet Infect Dis. 18:1127–1137. 30054190 10.1016/S1473-3099(18)30293-7

[20] Tani H , et al. 2016. Efficacy of T-705 (Favipiravir) in the treatment of infections with lethal severe fever with thrombocytopenia syndrome virus. mSphere. 1:e00061–15. 27303697 10.1128/mSphere.00061-15 PMC4863605

[21] Tani H , et al. 2018. Therapeutic effects of favipiravir against severe fever with thrombocytopenia syndrome virus infection in a lethal mouse model: dose-efficacy studies upon oral administration. PLoS ONE. 13:e0206416. 30365543 10.1371/journal.pone.0206416 PMC6203377

[22] Song R , Chen Z, Li W. 2020. Severe fever with thrombocytopenia syndrome (SFTS) treated with a novel antiviral medication, Favipiravir (T-705). Infection. 48:295–298. 31673977 10.1007/s15010-019-01364-9 PMC7223615

[23] Song P , et al. 2017. Downregulation of Interferon-beta and inhibition of TLR3 expression are associated with fatal outcome of severe fever with thrombocytopenia syndrome. Sci Rep. 7:6532. 28747721 10.1038/s41598-017-06921-6 PMC5529500

[24] Ding YP , et al. 2014. Prognostic value of clinical and immunological markers in acute phase of SFTS virus infection. Clin Microbiol Infect. 20:O870–O878. 24684627 10.1111/1469-0691.12636

[25] Sun Y , et al. 2012. Host cytokine storm is associated with disease severity of severe fever with thrombocytopenia syndrome. J Infect Dis. 206:1085–1094. 22904342 10.1093/infdis/jis452

[26] Matsuno K , et al. 2017. Animal models of emerging tick-borne phleboviruses: determining target cells in a lethal model of SFTSV infection. Front Microbiol. 8:104. 28194148 10.3389/fmicb.2017.00104 PMC5276813

[27] Yoshikawa R , Sakabe S, Urata S, Yasuda J. 2019. Species-specific pathogenicity of severe fever with thrombocytopenia syndrome virus is determined by Anti-STAT2 activity of NSs. J Virol. 93:e02226–18. 30814285 10.1128/JVI.02226-18 PMC6498056

[28] Dyer DP , et al. 2019. Chemokine receptor redundancy and specificity are context dependent. Immunity. 50:378–389.e5. 30784579 10.1016/j.immuni.2019.01.009 PMC6382461

[29] Liu J , et al. 2017. Dynamic changes of laboratory parameters and peripheral blood lymphocyte subsets in severe fever with thrombocytopenia syndrome patients. Int J Infect Dis. 58:45–51. 28249810 10.1016/j.ijid.2017.02.017

[30] Liu MM , Lei XY, Yu H, Zhang JZ, Yu XJ. 2017. Correlation of cytokine level with the severity of severe fever with thrombocytopenia syndrome. Virol J. 14:6. 28086978 10.1186/s12985-016-0677-1 PMC5237221

[31] Ning YJ , et al. 2019. Interferon-gamma-directed inhibition of a novel high-pathogenic phlebovirus and viral antagonism of the antiviral signaling by targeting STAT1. Front Immunol. 10:1182. 31191546 10.3389/fimmu.2019.01182 PMC6546826

[32] Tanaka T , Narazaki M, Kishimoto T. 2014. IL-6 in inflammation, immunity, and disease. Cold Spring Harb Perspect Biol. 6:a016295. 25190079 10.1101/cshperspect.a016295 PMC4176007

[33] Velazquez-Salinas L , Verdugo-Rodriguez A, Rodriguez LL, Borca MV. 2019. The role of interleukin 6 during viral infections. Front Microbiol. 10:1057. 31134045 10.3389/fmicb.2019.01057 PMC6524401

[34] Shimada S , Posadas-Herrera G, Aoki K, Morita K, Hayasaka D. 2015. Therapeutic effect of post-exposure treatment with antiserum on severe fever with thrombocytopenia syndrome (SFTS) in a mouse model of SFTS virus infection. Virology. 482:19–27. 25817401 10.1016/j.virol.2015.03.010 PMC7125729

[35] Liu Y , et al. 2014. The pathogenesis of severe fever with thrombocytopenia syndrome virus infection in alpha/beta interferon knockout mice: insights into the pathologic mechanisms of a new viral hemorrhagic fever. J Virol. 88:1781–1786. 24257618 10.1128/JVI.02277-13 PMC3911604

[36] Weber F , et al. 2002. Bunyamwera bunyavirus nonstructural protein NSs counteracts the induction of alpha/beta interferon. J Virol. 76:7949–7955. 12133999 10.1128/JVI.76.16.7949-7955.2002 PMC155133

[37] Bouloy M , et al. 2001. Genetic evidence for an interferon-antagonistic function of rift valley fever virus nonstructural protein NSs. J Virol. 75:1371–1377. 11152510 10.1128/JVI.75.3.1371-1377.2001 PMC114043

[38] Muralidharan A , Reid SP. 2021. Complex roles of neutrophils during arboviral infections. Cells. 10:1324. 34073501 10.3390/cells10061324 PMC8227388

[39] Peng C , et al. 2016. Decreased monocyte subsets and TLR4-mediated functions in patients with acute severe fever with thrombocytopenia syndrome (SFTS). Int J Infect Dis. 43:37–42. 26701820 10.1016/j.ijid.2015.12.009

[40] Qu B , et al. 2012. Suppression of the interferon and NF-kappaB responses by severe fever with thrombocytopenia syndrome virus. J Virol. 86:8388–8401. 22623799 10.1128/JVI.00612-12 PMC3421730

[41] Drescher B , Bai F. 2013. Neutrophil in viral infections, friend or foe?. Virus Res. 171:1–7. 23178588 10.1016/j.virusres.2012.11.002 PMC3557572

[42] Xu S , Cao X. 2010. Interleukin-17 and its expanding biological functions. Cell Mol Immunol. 7:164–174. 20383173 10.1038/cmi.2010.21 PMC4002915

[43] Griffin GK , et al. 2012. IL-17 and TNF-α sustain neutrophil recruitment during inflammation through synergistic effects on endothelial activation. J Immunol. 188:6287–6299. 22566565 10.4049/jimmunol.1200385 PMC3370121

[44] Kwon JS , et al. 2018. Kinetics of viral load and cytokines in severe fever with thrombocytopenia syndrome. J Clin Virol. 101:57–62. 29427908 10.1016/j.jcv.2018.01.017 PMC7106421

[45] Kwon J-S , et al. 2021. Viral and immunologic factors associated with fatal outcome of patients with severe fever with thrombocytopenia syndrome in Korea. Viruses. 13:2351. 34960620 10.3390/v13122351 PMC8703577

[46] Yoo JR , et al. 2021. IL-6 and IL-10 levels, rather than viral load and neutralizing antibody titers, determine the fate of patients with severe fever with thrombocytopenia syndrome virus infection in South Korea. Front Immunol. 12:711847. 34484214 10.3389/fimmu.2021.711847 PMC8416084

[47] Rubin EJ , Longo DL, Baden LR. 2021. Interleukin-6 receptor inhibition in Covid-19 – cooling the inflammatory soup. N Engl J Med. 384:1564–1565. 33631064 10.1056/NEJMe2103108 PMC7944949

[48] Garrison AR , Smith DR, Golden JW. 2019. Animal models for Crimean-Congo hemorrhagic fever human disease. Viruses. 11:590. 31261754 10.3390/v11070590 PMC6669593

[49] Caroline AL , Kujawa MR, Oury TD, Reed DS, Hartman AL. 2015. Inflammatory biomarkers associated with lethal rift valley fever encephalitis in the lewis rat model. Front Microbiol. 6:1509. 26779164 10.3389/fmicb.2015.01509 PMC4703790

[50] Gorman MJ , et al. 2018. An immunocompetent mouse model of Zika virus infection. Cell Host Microbe. 23:672–685.e676. 29746837 10.1016/j.chom.2018.04.003 PMC5953559

[51] Boring L , et al. 1997. Impaired monocyte migration and reduced type 1 (Th1) cytokine responses in C-C chemokine receptor 2 knockout mice. J Clin Invest. 100:2552–2561. 9366570 10.1172/JCI119798 PMC508456

[52] Brennan B , et al. 2015. Reverse genetics system for severe fever with thrombocytopenia syndrome virus. J Virol. 89:3026–3037. 25552716 10.1128/JVI.03432-14 PMC4337530

[53] Percie du Sert N , 2020. Reporting animal research: explanation and elaboration for the ARRIVE guidelines 2.0. PLOS Biol. 18:e3000411. 32663221 10.1371/journal.pbio.3000411 PMC7360025

