# Supplementary material for: Exploration of immunological responses underpinning severe fever with thrombocytopenia syndrome virus infection reveals IL-6 as a therapeutic target in an immunocompromised mouse model
Source: PNAS Nexus. 2022 Mar 10;1(1):pgac024. doi: 10.1093/pnasnexus/pgac024 (PMC9071185; doi:10.1093/pnasnexus/pgac024)
Supplement: pgac024_Supplemental_File [file pgac024_supplemental_file.pdf]

Supplementary Figures for

Exploration of immunological responses underpinning severe fever with thrombocytopenia syndrome reveals IL-6 as a therapeutic target.

Steven R. Bryden, James I. Dunlop, Andrew T. Clarke, Mazigh Fares, Marieke Pingen, Yan Wu, Brian J. Willett, Arvind H. Patel, George F. Gao, Alain Kohl & Benjamin Brennan*

* Corresponding Author: ben.brennan@glasgow.ac.uk

The PDF file includes:

Fig S1. Characterisation of SFTSV infection in a hSTAT2 knock in mouse model.

Fig S2. Pathological changes in the dLN and spleen following infection with SFTS viruses.

Fig S3. Prior inoculation with delNSs SFTSV induces protective adaptive immune response that is protective to subsequent lethal challenge in naïve mice.

Fig S4. Bone marrow derived DCs are susceptible to SFTSV infection.

Fig S5. Gating strategy used in flow cytometric analysis.

Fig S6. Clinical Scores for animals shown in Fig. 6A and 6B.

Table S1. Oligonucleotides used in this study.

Table S2. Scoring system for the welfare assessment of virus challenged experimental mice.

Table S3. List of antibodies used in these studies.

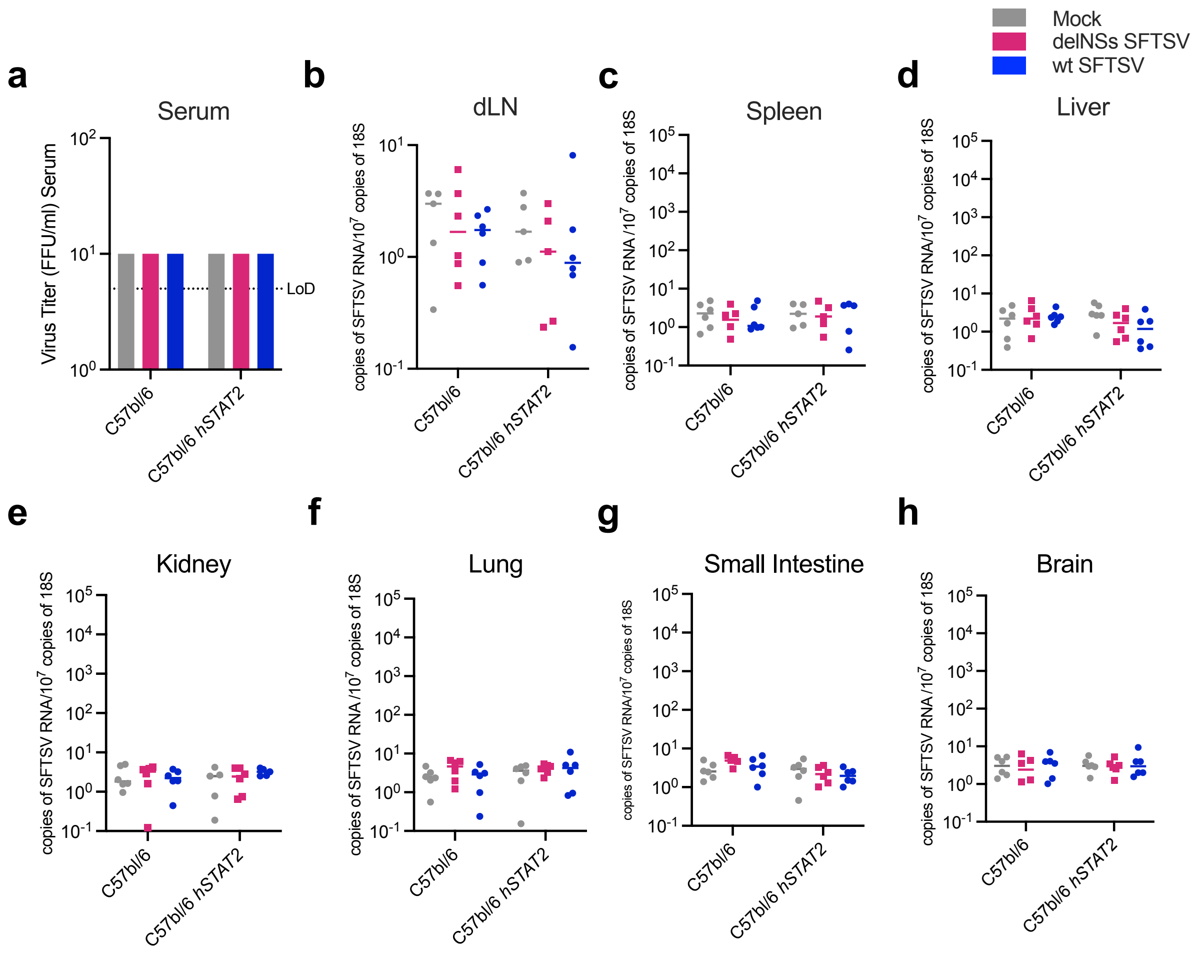

Fig S1. Characterisation of SFTSV infection in a hSTAT2 knock in mouse model. Groups of six hSTAT2 KI mice or control C57BL/6 mice were mock infected (grey) or inoculated with 10^5 foci-forming units (FFU) of delNSs (pink) or wt (blue) SFTSV. At PID 3, viral titres were assessed in the serum of infected animals by foci-forming assay **(a).** Tissues from mice infected with delNSs SFTSV (pink), wt SFTSV (blue) or mock-infected (grey) were collected at PID 3 and viral copy numbers were assessed in the draining lymph node (dLN) **(b)**, spleen **(c)**, liver **(d)**, kidney **(e)**, lung **(f)**, small intestine **(g)** and brain **(h)** by real-time PCR.

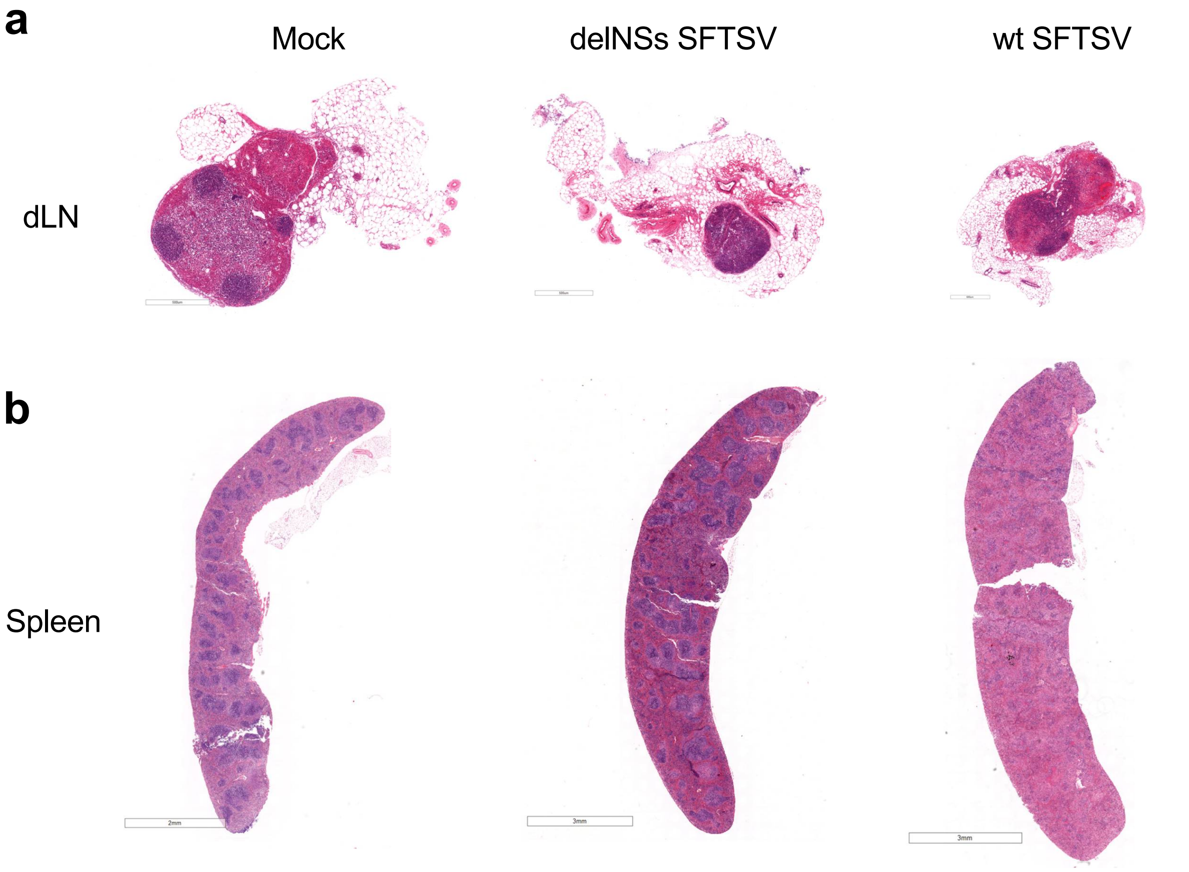

Fig S2. Pathological changes in the dLN and spleen following infection with SFTS viruses. A129 *IFNAR-/-* mice were inoculated with 10^5 foci-forming units (FFU) of wt or delNSs SFTSV or mock infected. At PID 3, animals were killed and lymph node **(a)** or spleen **(b)** of infected animals were processed for pathological examination by haematoxylin and eosin staining.

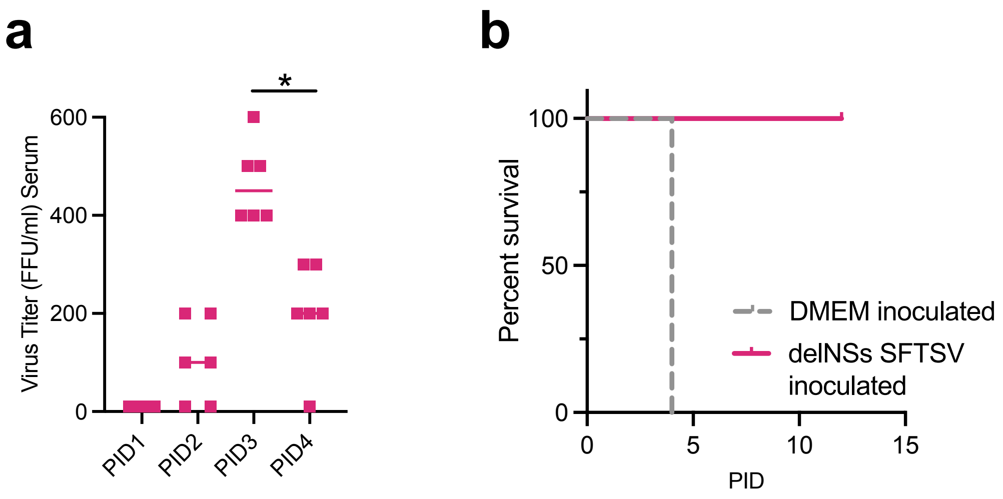

Fig S3. Prior inoculation with delNSs SFTSV induces protective adaptive immune response that is protective to subsequent lethal challenge in naïve mice. Groups of six A129 *IFNAR-/-* mice were inoculated with 10^5 foci-forming units (FFU) of delNSs- SFTSV (pink) or mock infected (grey). **(a)** Viral titre was assessed in the serum of infected animals at the indicated time point by foci-forming assay. At PID 14, animals were culled, and blood was collected. Total IgG was purified from the sera of mock or immunised mice and pooled together accordingly. A129 *IFNAR-/- mice* were administered 200 ug of pooled total IgG from either mock inoculated or delNSs SFTSV infected animals by intraperitoneal injection at PID -1. **(b)** All animals were then lethally challenged with 10^5 foci-forming units (FFU) of wt SFTSV at PID 0 and infection was allowed to progress until clinical signs were observed and humane endpoints were reached. Asterisks indicated significance * $P < 0.05$ as measured by a Wilcoxon matched pairs signed rank test **(a)**.

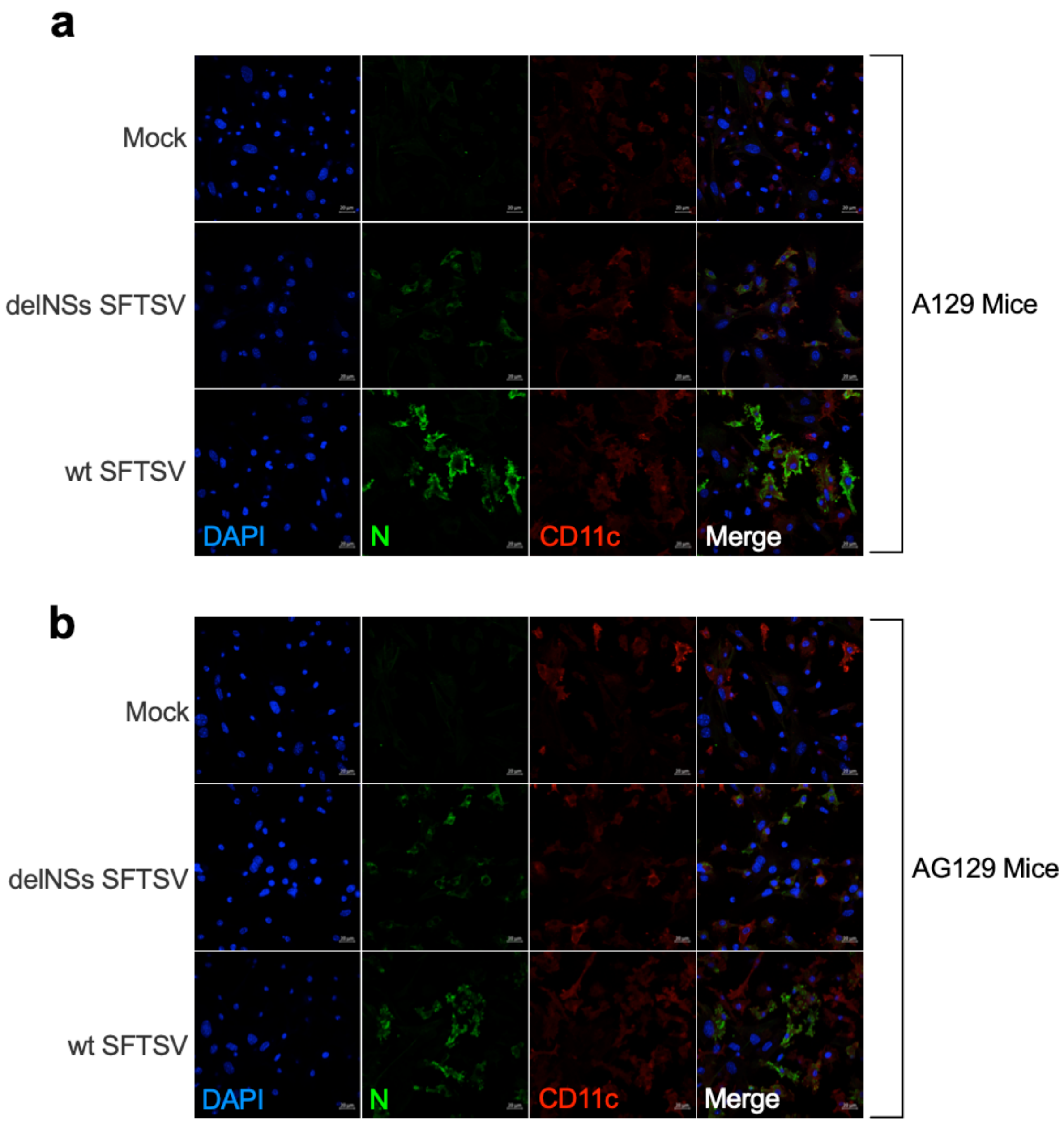

Fig S4. Bone marrow derived DCs are susceptible to SFTSV infection. Co-cultures of bone marrow derived GM-CSF dendritic cells and dermal fibroblasts from either A129 *IFNAR-/-* mice **(a)** or A129 *IFNAGR-/-* mice **(b)** were infected with delNSs SFTSV or wt SFTSV at a multiplicity of infection (moi) of 0.1 FFU/cell or mock infected. At 24 h p.i., cell monolayers were fixed in 4% formaldehyde, followed by staining with monospecific antibodies, rabbit anti-N (green) or mouse anti-CD11c (red) and cell nuclei stained with DAPI (blue).

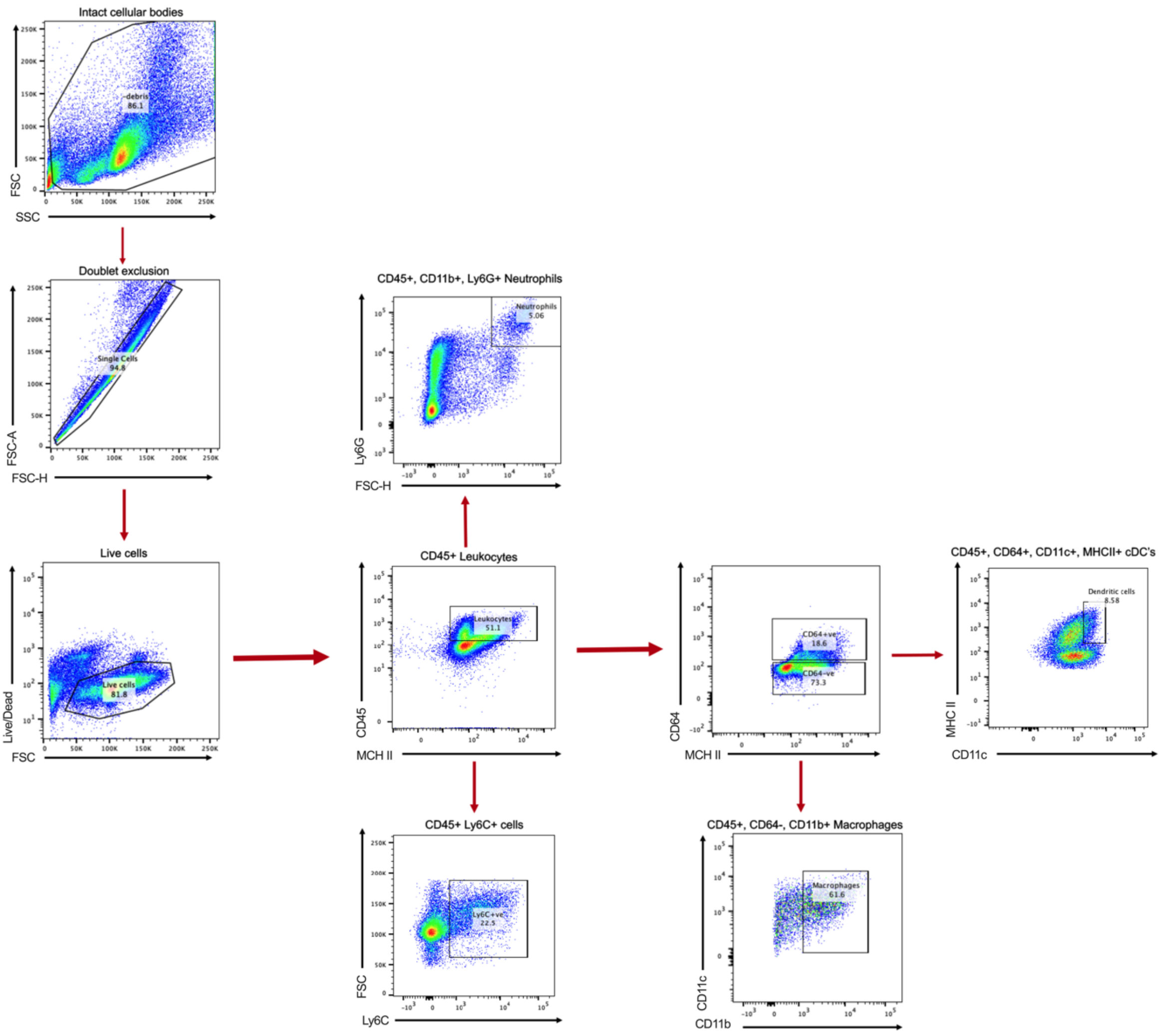

Fig S5. Gating strategy used in flow cytometric analysis. Exemplar of gating strategy for flow cytometric analyses used in this study.

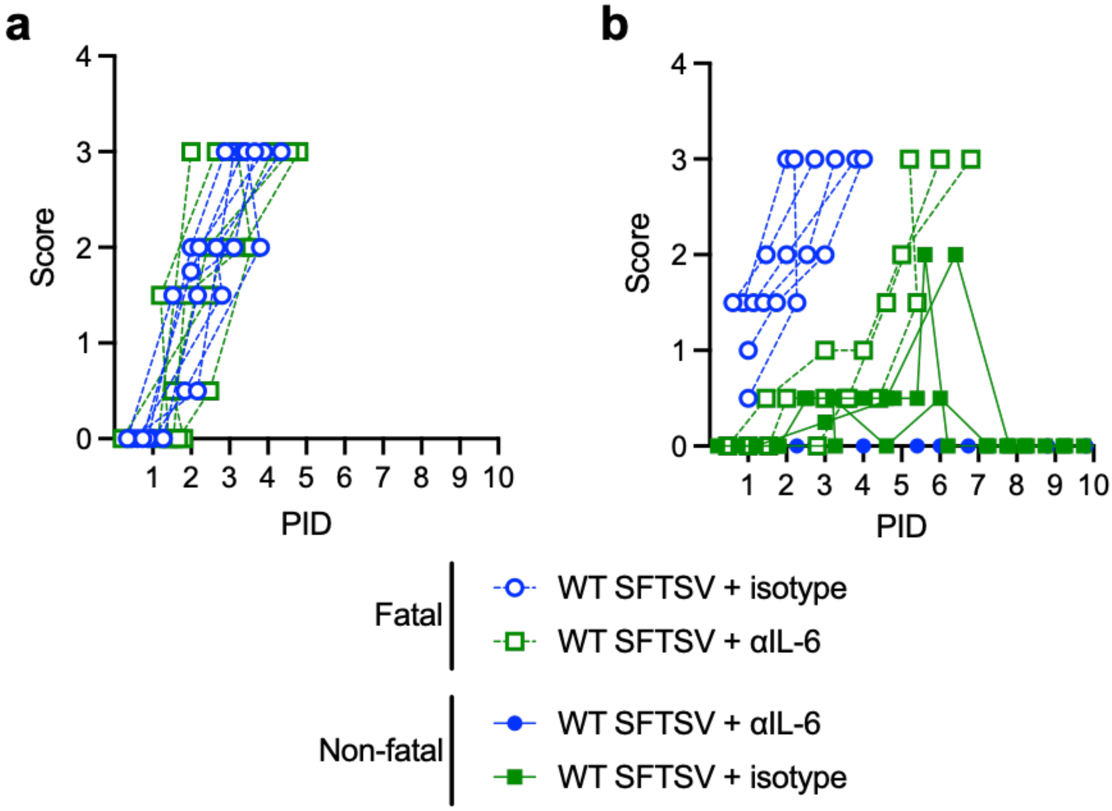

Fig S6. Clinical scores for animals shown in Fig. 6A and 6B. Groups of six A129 *IFNAR*$^{-/-}$ mice were interperitoneally administered an isotype control antibody (blue) or an anti-IL-6 antibody (green), either prophylactically at PID -2 **(a)** or therapeutically at PID 2 **(b).** Clinical scores are presented for each individual animal over time. Open shapes indicate a mouse with a fatal outcome following infection, closed shapes indicate the animal survived the infection until the endpoint of the study at PID 10.

Table S1. Oligonucleotides used in this study. List of the oligonucleotide primers used for qPCR analysis of immune genes.

Oligo Name	Sequence 5' – 3'
IL-10 qpcr 1	CAGTACAGCCGGGAAGACAA
IL-10 qpcr 2	TTCTGGGCCATGCTTCTCTG
IL-12α qpcr1	GGTGAAGACGGCCAGAGAAA
IL-12α qpcr2	CCAAGGCACAGGGTCATCAT
18S qpcr1	GACTCAACACGGGAAACCTC
18s qpcr2	TAACCAGACAAATCGCTCCAC
TNFα qpcr1	CACCACCATCAAGGACTCAA
TNFα qpcr2	GAGGCAACCTGACCACTCTC
CCL2 qpcr1	CTCACCTGCTGCTACTCATTCA
CCL2 qpcr2	CCATTCCTTCTTGGGGTCA
Rsad2 qpcr1	TGAAGCGTGGCGGAAAGTAT
Rsad2 qpcr2	TCCTTCCCATCTCAGCCTCA
IL-1β qpcr1	CGCTCAGGGTCACAAGAAAC
IL-1β qpcr2	GAGGCAAGGAGGAAAACACA
IFNɣ qpcr1	AGCAAGGCGAAAAAGGATG
IFNɣ qpcr2	CTGGACCTGTGGGTTGTTG
IFNα4 qpcr1	GCAATGACCTCCATCAGCA
IFNα4 qpcr2	GTATGTCCTCACAGCCAGCA
IFNβ qpcr1	CACAGCCCTCTCCATCAACT
IFNβ qpcr2	GCATCTTCTCCGTCATCTCC
CXCL10 qpcr1	GCTCAAGTGGCTGGGATG
CXCL10 qpcr2	GAGGACAAGGAGGGTGTGG
SFTSV genome 1	AGCCTTCTTCACGACAAGCA
SFTSV genome 2	TTCGTCATGGCTCAGGAACC
IL-6 qpcr 1	TTCCATCCAGTTGCCTTCTT
IL-6 qpcr 2	ATTTCCACGATTTCCCAGAG
IL-12 qpcr 1	CCAAACCAGCACATTGAAGA
IL-12 qpcr 2	CTACCAAGGCACAGGGTCA
IL-17 qpcr 1	ATGGCAAGGAACCTGTGGAG
IL-17 qpcr 2	GAGAACACTTCCTGGGACGG

Mild (Score 1)	Moderate (Score 2)	Advanced (Score 3)
Reduced weight gain	Weight loss between 10-15%	Weight loss greater than 15%
Slight unkempt appearance	Piloerection	Staring coat-marked piloerection
Subdued but responsive, animal shows normal provoked patterns of behaviour	Subdued animal shows subdued behaviour patterns	Unresponsive to extraneous activity and provocation
Hunched only occasionally	Hunched intermittently	Hunched persistently/markedly hunched
Very slight alteration in respiration	Intermittent abnormal breathing	Laboured respiration
Eyes slightly dull, not bright	Semi-closed eyelids	Closed eyes most of the time
Interacts with peers	Reduced peer interaction	Very little or no interaction with peers/dull
Slight gait alteration	Intermittent tremors/incoordination OR loss of one limb function	Persistent (>24 h) tremors/incoordination OR paresis OR paraplegia
	Transient prostration (lying stretched out)/excitement (<1 h)	Prolonged prostration (lying stretched out) / excitement (>1 h)
	Sporadic loose stools	Frank diarrhoea
		Loss of bladder function
		Self-mutilation

Table S2. Scoring system for the welfare assessment of virus challenged experimental mice.

Antigen (anti-)	Clone	Isotype	flurophore	Company	Catalogue No
GFP	FM264G	Rat IgG	APC	BioLegend	338010
CD11b	M1/70	Rat IgG	APC	BD Biosciences	557396
CD11c	N418	Armenian hamster IgG	eflour615	Invitrogen	42-0114-82
CD11c	N418	Rat IgG	Pe	BioLegend	117308
CD11c	N418	Armenian hamster IgG	BV711	BioLegend	117349
CD11c	N418	Armenian hamster IgG	APC	BioLegend	117310
CD11c	EPR21826	Rabbit IgG	N/A	Abcam	ab219799
CD19	6D5	Rat IgG	BV421	BioLegend	115537
CD19	1D3	Rat IgG	FITC	BioLegend	152404
CD3	17A2	Rat IgG	PerCP/cy5.5	BioLegend	100218
CD45	30-F11	Rat IgG	Pe/Cy5	BioLegend	103109
CD45	30-F11	Rat IgG	PerCP/cy5.5	BioLegend	103130
CD61	2C9.G2	Armenian hamster IgG	Pe	BioLegend	104307
CD64	X54-5/7.1	Mouse IgG	BV421	BioLegend	139309
CXCR2	SA044G4	Rat IgG	Pe/Cy7	BioLegend	149315
F5/80	BM8	Rat IgG	Pe/Cy7	Invitrogen	25-4801-82
I-Ab (MHC II)	AF6-120.1	Mouse	Pe/Cy7	BioLegend	116419
Ly-6C	HK1.4	Rat IgG	Pe	BioLegend	128007
Ly-6C	HK1.4	Rat IgG	Pe/Cy7	BioLegend	128017
Ly-6C	HK1.4	Rat IgG	AF700	BioLegend	128024
Ly-6C	ER-MP20	Rat IgG	Biotin	Abcam	ab15674
ly-6G	1A8	Rat IgG	FITC	BioLegend	127606
MHC II	M5/114.15.2	Rat IgG	BV605	BioLegend	107639
Siglec F	E50-2440	Rat IgG	Pe	BD Biosciences	552126
Streptavidin	n/a	n/a	BUV395 strep	BD Biosciences	564176
N	SFTSV (HB29)	Rabbit IgG		In House	https://doi.org/10.1128/jvi.03432-14
HRP	heavy and light chain	Rabbit IgG		Cell Signalling	7074
IL-6	MP5-20F3	Rat IgG		2B Scientific	BE0046
Trinitrophenol	2A3	Rat IgG		BioXCell	BP0089

Table S3. List of antibodies used in these studies.
